# Marine Sponge Endosymbionts: Structural and Functional Specificity of the Microbiome within *Euryspongia arenaria* Cells

**DOI:** 10.1128/spectrum.02296-21

**Published:** 2022-05-02

**Authors:** Qi Yang, Jackson K. B. Cahn, Jörn Piel, Yue-Fan Song, Wei Zhang, Hou-Wen Lin

**Affiliations:** a Center for Marine Drugs, State Key Laboratory of Oncogene and Related Genes, Department of Pharmacy, Renji Hospital, School of Medicine, Shanghai Jiao Tong University, Shanghai, China; b Centre for Marine Bioproducts Development, College of Medicine and Public Health, Flinders University, South Australia, Australia; c Institute of Microbiology, Eidgenössische Technische Hochschule (ETH) Zurich, Zurich, Switzerland; d College of Food Science and Engineering, Key Laboratory of Aquatic Product Processing and Utilization of Liaoning Province, Dalian Ocean University, Dalian, China; University Of Thessaly

**Keywords:** sponge *Euryspongia arenaria*, sponge cells, endosymbiotic microbiome, functional specificity, structural specificity

## Abstract

Sponge microbiomes are typically profiled by analyzing the community DNA of whole tissues, which does not distinguish the taxa residing within sponge cells from extracellular microbes. To uncover the endosymbiotic microbiome, we separated the sponge cells to enrich the intracellular microbes. The intracellular bacterial community of sponge *Euryspongia arenaria* was initially assessed by amplicon sequencing, which indicated that it hosts three unique phyla not found in the extracellular and bulk tissue microbiomes. These three phyla account for 66% of the taxonomically known genera in the intracellular microbiome. The shotgun metagenomic analysis extended the taxonomic coverage to viruses and eukaryotes, revealing the most abundant signature taxa specific to the intracellular microbiome. Functional KEGG pathway annotation demonstrated that the endosymbiotic microbiome hosted the greatest number of unique gene orthologs. The pathway profiles distinguished the intra- and extracellular microbiomes from the tissue and seawater microbiomes. Carbohydrate-active enzyme analysis further discriminated each microbiome based on their representative and dominant enzyme families. One pathway involved in digestion system and family esterase had a consistently higher level in intracellular microbiome and could statistically differentiate the intracellular microbiome from the others, suggesting that triacylglycerol lipases could be the key functional component peculiar to the endosymbionts. The identified higher abundance of lipase-related eggNOG categories further supported the lipid-hydrolyzing metabolism of endosymbiotic microbiota. Pseudomonas members, reported as lipase-producing bacteria, were only in the endosymbiotic microbiome, meanwhile Pseudomonas also showed a greater abundance intracellularly. Our study aided a comprehensive sponge microbiome that demonstrated the taxonomic and functional specificity of endosymbiotic microbiota.

**IMPORTANCE** Sponges host abundant microbial symbionts that can produce an impressive number of novel bioactive metabolites. However, knowledge on intracellular (endosymbiotic) microbiota is scarce. We characterize the composition and function of the endosymbiotic microbiome by separation of sponge cells and enrichment of intracellular microbes. We uncover a noteworthy number of taxa exclusively in the endosymbiotic microbiome. We unlock the unique pathways and enzymes of endosymbiotic taxa. This study achieves a more comprehensive sponge microbial community profile, which demonstrates the structural and functional specificity of the endosymbiotic microbiome. Our findings not only open the possibility to reveal the low abundant and the likely missed microbiota when directly sequencing the sponge bulk tissues, but also warrant future in-depth exploration within single sponge cells.

## INTRODUCTION

Marine sponges (phylum Porifera), which evolved ca. 750 million years ago, are the oldest extant metazoans (1). There are 9,490 valid species reported from marine and freshwater environments to date, which are found across tropical, temperate, and polar regions (2). Sponges are the most prolific sources of marine natural products, contributing almost 30% of all marine natural products reported during 2001 to 2010 (3). On average, about 200 to 350 new compounds were discovered from sponges each year since 2011 (4–13). The sponge species belonging to genus *Euryspongia* selected in this study is considered as a promising source of bioactive natural products since the initial report of euryfuran production (14). A new cyclized 9,11-secosterol enol-ether was first isolated from South Australian sponge *E. arenaria* (15). Various secondary metabolites with different bioactivities, such as cytotoxic, antifungal, antihistaminic, anti-HIV, and anti-cancer activities (16–24), have subsequently been reported from sponges belonging to genus *Euryspongia*.

Sponges form symbiotic relationships with often complex communities of microorganisms (25–30). Up to 40% to 60% of the tissue volume of certain sponge species can consist of microorganisms at a density exceeding 10^9^ microbial cells per mL of sponge tissue, orders of magnitude greater than concentrations in surrounding seawater or sediment (31–33). The associated microbes produce diverse metabolites that have stimulated the interest of the pharmaceutical industry (34–38). Typically, the prokaryotic composition of sponge microbiomes is analyzed by high-throughput 16S rRNA gene-based sequencing approaches (27–30, 39–43). Recently, metagenomic sequencing provided new insights into the functional gene repertoires of sponges and the metabolic pathways carried by sponge symbionts (26, 44–46). However, metagenomic methods can be challenging in samples with low-microbial abundance or in those, like sponges, dominated by host DNA (47). For the members of a microbiome not yet characterized by culture-based methods, a significant fraction of reads in the metagenome may still remain unmatched after assembly (48). The quality of assembly can be impacted by the complexity of the community and the sequencing technology (49).

Sponge microbiomes are predominantly studied via directly sequencing the community DNA of whole tissue samples. They have shown host specificity and remarkable stability across a large range of environmental conditions (25, 27, 29, 41, 43), although for certain sponge species, shifts in community structure under different biogeographic locations and seasons have been observed (50–55). A pretreatment to enrich extracellular prokaryotes before DNA isolation has also been employed for the investigation of sponge microbiomes (44, 56–60). However, knowledge on intracellular sponge microbiota is scarce (61–64) and only two types of endosymbiotic bacteria have been identified—calcifying bacteria (Calcibacteria) (61–63) and renieramycin-producing bacteria (*Candidatus* Endohaliclona renieramycinifaciens) (64). Of particular interest are the endosymbiotic microbiomes of archaeocytes and choanocytes, which constitute the stem cell population in all four classes of sponges and generate all cell types for reconstituting dissociated tissues (65).

Our study aims to reveal the sponge cell endosymbiotic microbiome by sequencing the metagenomic DNA isolated from the sponge cells after enrichment and purification, as well as to profile its functional specificity compared to the extracellular, whole tissue, and seawater microbiomes. A South Australia locally growing *Euryspongia arenaria* was selected as the model sponge in our study because this sponge could be maintained in a controlled aquarium system under stable living conditions in pilot experiments of sponge aquaculture and sponge cell separation. An optimized sponge cell purification strategy was utilized to collect the sponge cell fractions. A newly developed multi-primer amplicon sequencing approach was applied to profile the microbial communities. Finally, shotgun metagenomic sequencing was further employed to investigate the functional gene diversity specific to the endosymbiotic microbiome. With this study, we provide a comprehensive profile of the *E. arenaria* microbiome to demonstrate the specificity of its intracellular microbial community regarding structural and functional features. Metagenome based annotations allowed for a correlation analysis between the functions and their potential microbial origins.

## RESULTS

### Enrichment of sponge cells and extracellular bacteria.

**FIG 1:**
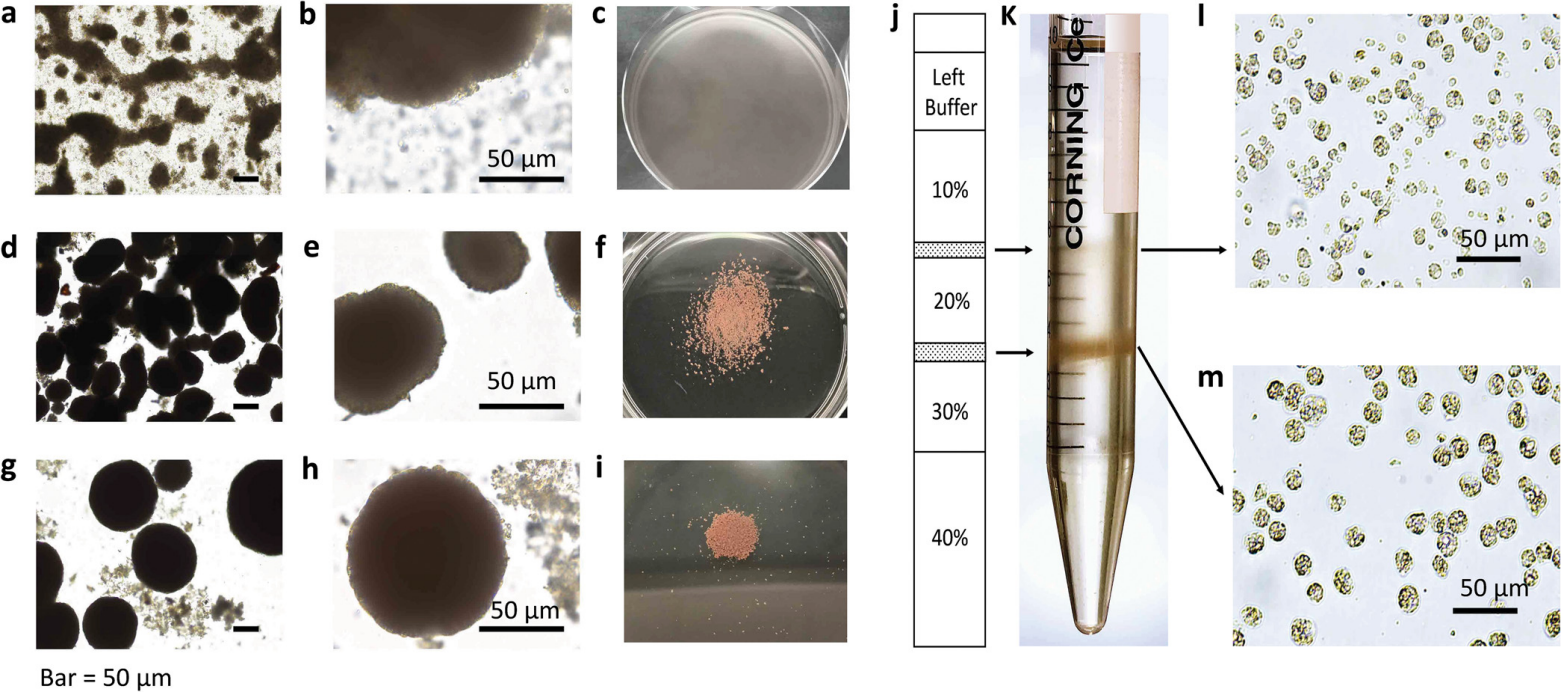
Sponge cells cultivation and purification. (a, b) Aggregated sponge cells by *in vitro* cultivation in artificial seawater for 2 h, vigorous cells started gathering. (c) Cells were inoculated in a Petri dish with artificial seawater after 2 h. (d, e) 6 h cultivation, vigorous cells formed solid aggregates. (f) Cells were inoculated in a Petri dish with artificial seawater after 6 h. (g, h) 12 h cultivation, aggregates became bigger in size. (i) Cells were cultivated in a Petri dish with artificial seawater after 12 h. (j) Illustration of Ficoll-CMFASW density gradient and cell fractions after centrifugation. (k) Ficoll-CMFASW density gradient and cell fractions after centrifugation in tube. (l) Optical microscopy image of sponge cell fraction between 10% and 20% Ficoll-CMFASW gradients. (m) Optical microscopy image of sponge cell fraction between 20% and 30% Ficoll-CMFASW gradients.

To investigate intra- and extracellular microbiomes, sponge tissues were first dissociated for target fraction enrichment. Sponge archaeocytes (triangle, Fig. S1a and b) and choanocytes (circle, Fig. S1a and b) were enriched at 100 × g centrifugation (Fig. S1c) to separate out most of the extracellular bacteria. The microbial population was enriched gradually in the pellets as the centrifugation speed increased (Fig. S1d to f). The pellets from the centrifugations at 2,000 × g and 16,000 × g were combined to profile the extracellular bacteria community. The enriched sponge cells were further purified by *in vitro* cultivation in artificial seawater for 12 h (Fig. 1) until the aggregates formed “primmorphs” (66) with smooth surfaces (Fig. 1g and h). An additional gradient density centrifugation step separated two cell fractions: one was enriched in archaeocyte cells, comprising the band between 20% and 30% Ficoll (Fig. 1l), and the other was enriched in choanocyte cells with some epithelial cells present, comprising the 10% to 20% Ficoll layer (Fig. 1m). Each fraction was enriched by more than 70% for individual cell types. DNA sequence analysis was carried out on these two purified sponge cell fractions to reveal the microbial community of the archaeocyte- and choanocyte-dominant fraction, respectively.

### Ultrastructural characterization of sponge cells.

**FIG 2:**
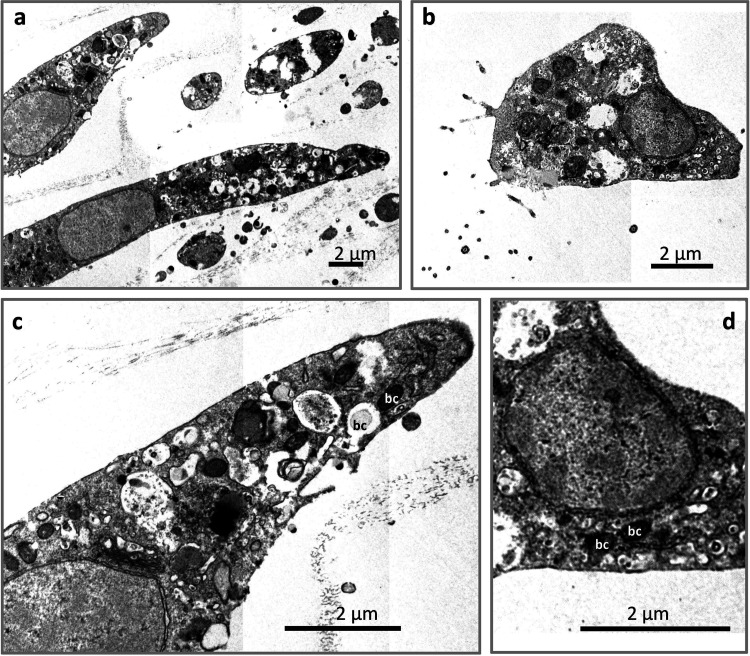
Morphological characterization of sponge cells and endosymbiotic microbe identification by transmission electron microscopy. (a) Archaeocytes. (b) Choanocytes. (c) Endosymbiotic bacteria-like cells within one archaeocyte cell shown in a. (d) Endosymbiotic bacteria-like cells within one choanocyte cell shown in b. The white “bc” highlights the non-digested bacteria like cells, the black “bc” highlights the digested bacteria like cells.

To characterize sponge cell types, transmission electron microscopy (TEM) was utilized to observe and measure cells. The typical archaeocytes in the purified fraction were usually 10 μm or above in diameter (Fig. 2a) with prominent and solid lysosomes and large nuclei, which were 3 μm or above in diameter. The choanocytes, usually less than 8 μm in diameter (Fig. 2b), were generally smaller than archaeocytes. In fixed tissues, the choanocytes were surrounded by cilia. Vacuoles were frequently found in archaeocytes with digested bacteria inside (Fig. 2c). In addition, bacteria-like cells were detected to be scattered in the cytoplasm of both archaeocytes (Fig. 2c) and choanocytes (Fig. 2d). The most observed bacteria-like organisms are 0.5 μm long and rod-shaped with condensed contents.

### Purity validation by consistent Raman spectra of individual sponge cells.

**FIG 3:**
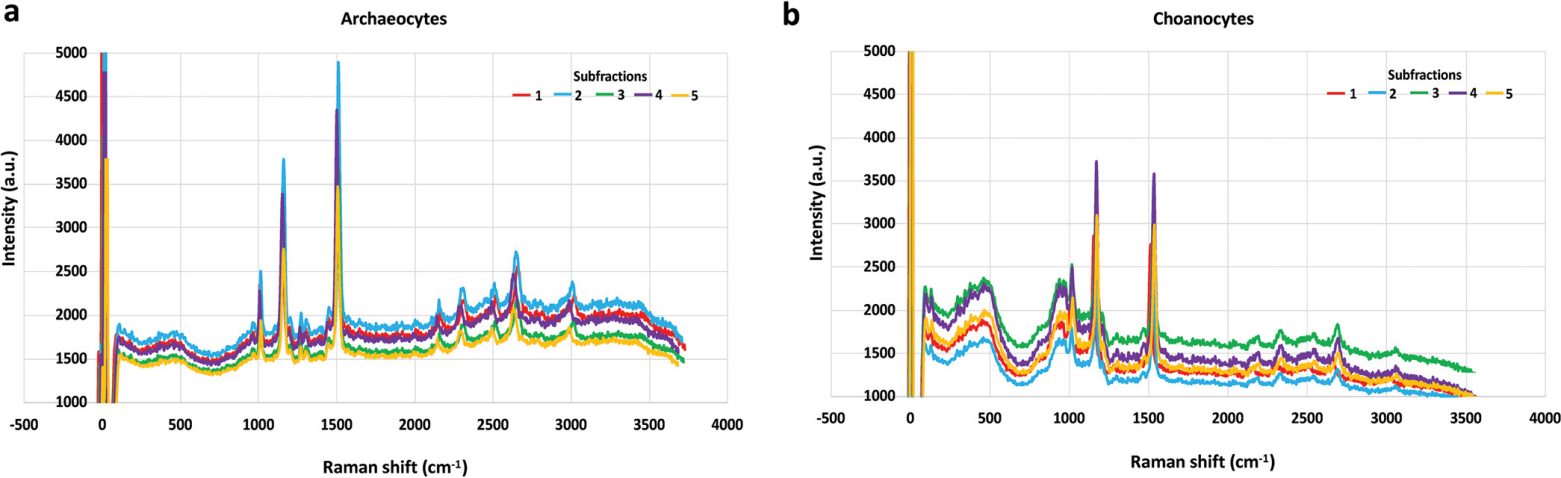
Raman spectra of single sponge cell for validation of homogenous cell sorting. (a) Spectra of each of 100 cells from five subfractions of purified sponge archaeocyte fraction. (b) Spectra of each of 100 cells from five subfractions of purified sponge choanocyte fraction. The combination is generated after normalization of zero peak.

To evaluate and validate the homogeneity of the sponge cells within the same fraction, *in situ* non-destructive detection by Raman spectroscopy was applied to directly characterize and compare the metabolite profile of individual living sponge cells. The observed fingerprint bands pointed to the presence of certain chemical components and intensity in the spectra in each sponge cell and its symbionts. The observed spectra of sponge cells in archaeocyte fraction are composed of three major bands located at 1,010 ± 5 cm^−1^, 1,151 ± 8 cm^−1^, and 1,508 ± 6 cm^−1^ (Fig. 3a). The band pattern of sponge choanocyte fraction is 1,008 ± 8 cm^−1^, 1,148 ± 6 cm^−1^, and 1,541 ± 8 cm^−1^ (Fig. 3b). The trends of the intensity ratio are different between the two cell fractions (Fig. 3). The Raman spectra of four randomly selected cells in each of five investigation areas within the same subfraction showed a highly consistent band pattern and intensity ratios. The spectra obtained by investigating five representative areas showed a stable consistency. Only slight variation between the subfractions was observed regarding the actual intensity of the major peaks.

### Microbial community compositions of different sponge cell types assessed by 16S rRNA gene amplicon sequencing.

**FIG 4:**
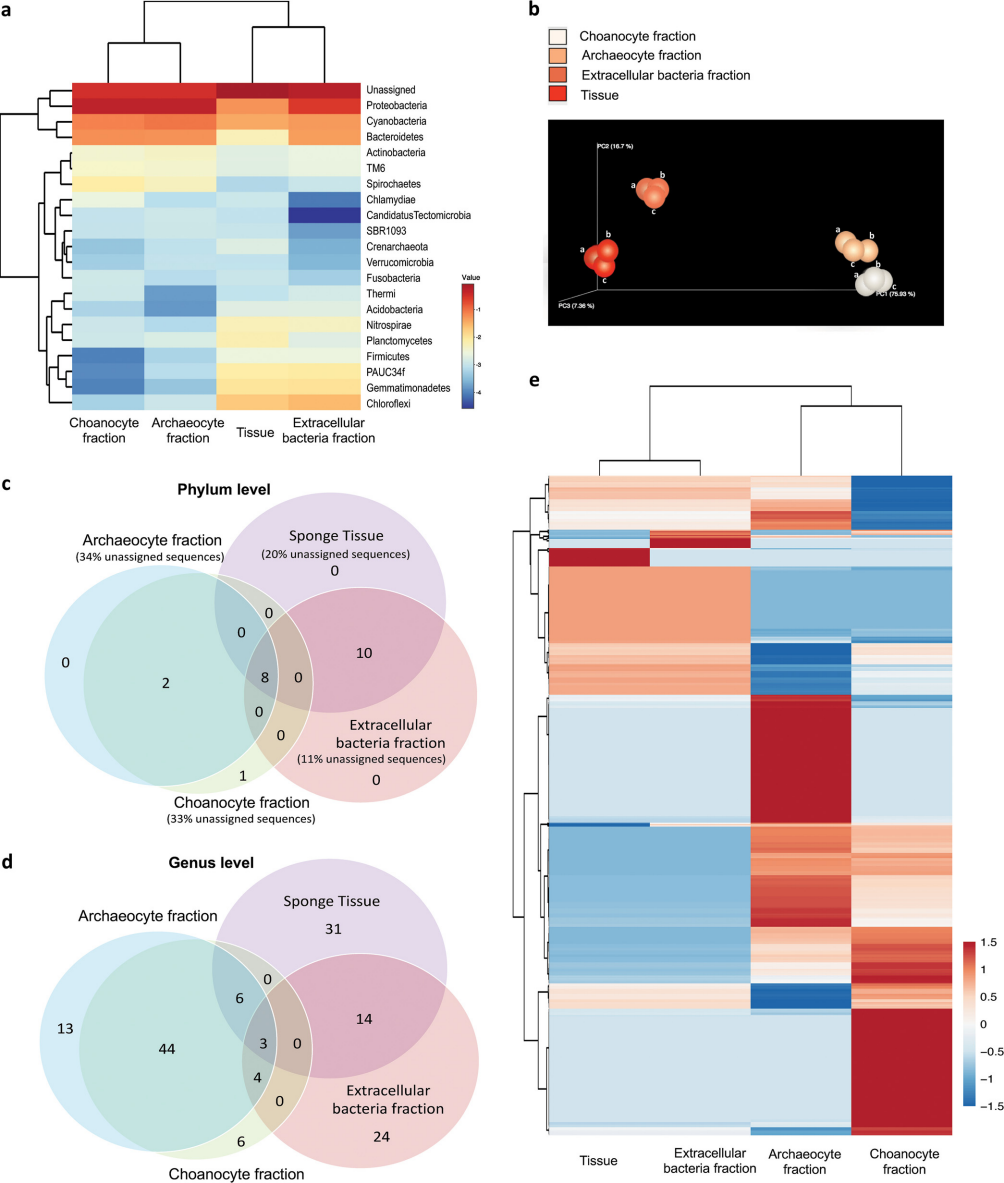
Comparisons among the four microbial communities revealed by amplicon analysis. (a) Taxonomic heatmap for phylum-level OTUs. (b) Principal coordinates analysis (PCoA) plot derived from Bray-Curtis distance among microbial communities (*P* = 0.0001 by PERMANOVA). Labels a to c distinguish the three biological replicates. (c) Venn diagram for microbial phylum level OTUs distribution. (d) Venn diagram for microbial genus level OTUs distribution. All the unclassified genus-level OTUs are counted as one. (e) Abundance of unique sequences in the four microbial communities. Data in heatmaps are centered and the unit scaling is applied. Both rows and columns are clustered using maximum distance method. The value of the color bar is calculated by log_10_ of the relative abundance of each OTU. The red and blue colors reflect the highest and lowest relative abundance in the community, respectively.

The 16S rRNA gene amplicon sequencing data covering the V1 to V8 regions revealed the sponge microbial communities of the prepared four types of samples. On average, sequence reads of 225,908 (< 0.08% variation between biological replicates), 544,449 (< 0.02% variation), 601,804 (< 0.04% variation), and 543,060 (< 0.06% variation) were obtained from the sponge tissue sample, archaeocyte fraction, choanocyte fraction), and the extracellular bacteria fraction, respectively (Table S1a). Rarefactions using three metrics (PD whole tree, Chao1, and observed species) demonstrated the archaeocyte fraction and choanocyte fraction had higher microbial richness compared to the other two communities (extracellular bacterial fraction and tissue sample) (Supplemental file 1). The curves generated using Shannon index of all samples already reached a plateau at this sequencing depth, suggesting that the sequencing was deep enough (Supplemental file 1). Combined, 21 phylum-level microbial OTUs, including 17 phyla, three candidate phyla, and one unassigned OTU, were revealed. The comparison between the communities based on the relative abundance of each OTU is provided as a hierarchical clustering heatmap in Fig. 3a. The dendrogram on top, obtained by using a “correlation” distance measurement and the “complete linkage” clustering method (67), indicated that the two purified sponge cell fractions displayed a similar community composition and structure (reflected by the relative abundance of each microbial taxon in the community) that were highly distinct from those of the sponge tissue and the enriched extracellular bacteria fraction (Fig. 4a). Moreover, the beta diversity using Bray-Curtis distance among the four microbial communities (*P* = 0.0001 by PERMANOVA) further supported the grouping pattern (Fig. 4b).

Both the whole-sponge tissues and the extracellular bacterial fraction showed higher microbial diversity of phylum-level OTUs than either cell fraction (Fig. 4c). However, members of three microbial phyla, Chlamydiae, Spirochaetes, and *Candidatus* Tectomicrobia, were only found in the intracellular cell fractions (Table S1b). Chlamydiae members were only obtained from the sponge archaeocytes. Sequencing of whole sponge tissue and bacteria-enriched fractions revealed the same microbial phyla, with 10 unique phyla (nine known phyla and one candidate phylum) compared with the sponge cell fractions. Eight phyla were commonly shared among the microbial communities of the whole sponge tissue and the three fractions.

A comparison at genus level (affiliated and candidate genera) of the microbial communities demonstrated that the sponge tissue contributed the highest number of unique known (candidate) genera, i.e., 31, compared with 24 found in the bacteria-enriched fraction and 19 from the two sponge cell fractions (Fig. 4d and Table S1c). Within the cell fractions, the archaeocyte fraction shared 57 known genera with the choanocyte fraction, while these two fractions had 13 and six unique genera, respectively. Only three genera were found present in all tested samples.

### Unique 16S rRNA gene sequence distribution among different types of sponge cell.

To go beyond taxonomic assignments, the distribution of unique sequences among the four sample types were investigated to compare their microbial communities. Based on a single-nucleotide difference cut-off for distinctive sequence identification, a total of 500 unique sequences were detected. Among them, 118 sequences could be assigned at the order level, but belong to an unidentified family; 382 sequences could be assigned to a known family; and 139 sequences could be assigned down to the genus level (Supplemental file 1). Notably, sequences assigned to the genus *Legionella*, a group of Gram-negative bacteria that includes human pathogens, were only detected in the extracellular bacteria fraction and the tissue sample (Supplemental file 1). A comparison between the communities is shown in a heatmap using hierarchical clustering analysis based on the relative abundance of the unique sequences inferred from raw data (Fig. 4e). The distribution of the unique sequences was found to be specific to each of the four microbial communities (Fig. 4e and Supplemental file 1).

### In-depth microbiota identification from different types of sponge cell.

**FIG 5:**
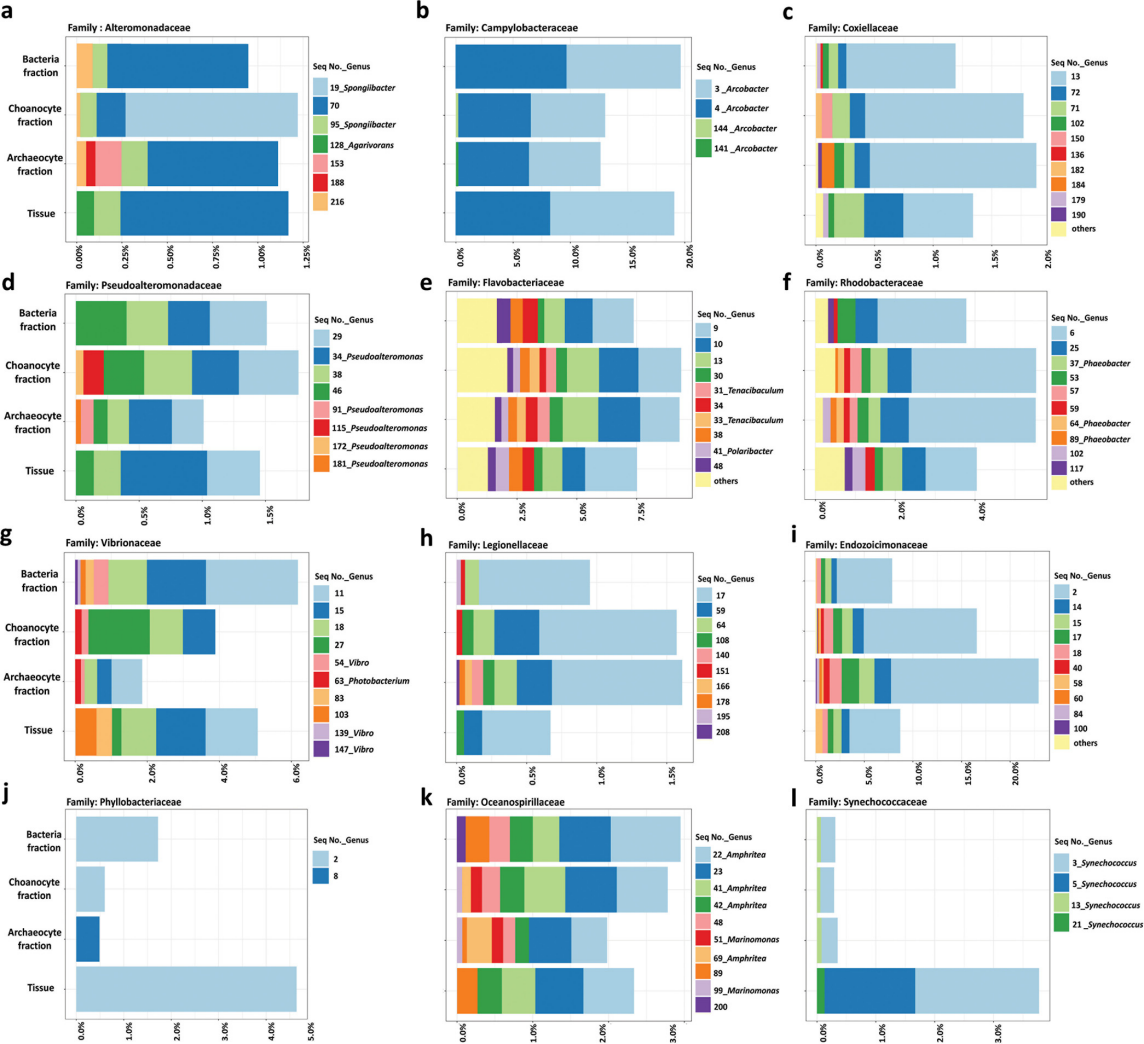
Distribution of multiple sequences within the same family or genus revealed by amplicon analysis. The title of the bars is labeled in one of the three subfigures sorted in the same row (i.e., subfigures a, d, g, and j). The other subfigures (b, c, e, f, h, i, k, and l) follow the same vertical axis format. The titles of each subfigure are the family names. Bacteria fraction, extracellular bacteria enriched; Tissue, whole sponge sample.

Using the unique sequences analysis approach, an in-depth characterization of the identified microbiotas was conducted to further distinguish the microbiomes at the sequence level. The in-depth comparison of multiple16S rRNA gene sequences assigned to the same genus or family allows differentiation between organisms at the species/subspecies level or inference of potential new species. Twelve families were detected that occurred in all the microbial communities of sponge tissue, purified sponge cell fractions, and the enriched extracellular bacteria fraction (Fig. 5 and Supplemental file 2), although distributions and abundances within these families varied across the four microbial communities. Additionally, the unique sequences specific to the microbial community of each of the cell fractions were identified. For the families of Alteromonadaceae, Campylobacteraceae, Coxiellaceae, and Pseudoalteromonadaceae, both sponge cell fractions had distinguishable unique sequences that were not present in the extracellular bacterial fraction and tissue sample (Fig. 5a to d). For Flavobacteriaceae, Rhodobacteraceae, and Vibrionaceae, the cell fractions shared unique sequence entries (Fig. 5e to g). For Legionellaceae (Fig. 5h), the unique sequences came from archeocyte fraction and the extracellular bacteria fraction. Moreover, for Endozoicomonadaceae, Phyllobacteriaceae, Oceanospirillaceae, and Synechococcaceae, the archeocyte fraction (Fig. 5i and j), extracellular bacteria fraction (Fig. 5k), or the tissue sample (Fig. 5l) were indicated as the single contributor for the unique sequence(s).

To explore potential novel microbiota from the unique sequences specific to individual sponge cell fractions, these multiple sequences belonging to the same families/genera were further distinguished at the genus/species level by conducting individual BLAST searches in the NCBI gene database. Moreover, the base variations between the sequences assigned as the same family/genus were observed. For example, the sequences (seq3, seq13, and seq21) assigned to genus *Synechococcus* in family Synechococcaceae were further inferred to match the species *Synechococcus rubescens* with similarity of 98%, 96%, and 96%, respectively. These in-depth classifications and the phylogenetic trees (Supplemental file 2) could identify potentially novel microbiota.

### Community structure specificity of the sponge intracellular microbiome uncovered by metagenome sequencing.

16S rRNA gene amplicon sequencing is useful for the identification of microbial community members, but shotgun metagenomic sequencing is required to identify the functional potential of a community by a marker gene approach. Considering the challenge of metagenomics in samples dominated by host DNA (Fig. S2), we removed the sequences belonging to sponges (Phylum Porifera) using the Burrows-Wheeler Aligner (BWA) before further processing. To better present the microbial community, the sequences of other eukaryotes except fungi were also filtered out for the rest of the analyses. As a result, an average of 70.2 million paired-end reads (150 bp) were collected for each sample (Supplemental file 3). After quality check, more than 80% reads were retained for *de novo* assembly (contigs or scaffolds). Approximately 4.1 million metagenes were then clustered into 2,442,690 non-redundant genes (unigenes).

**FIG 6:**
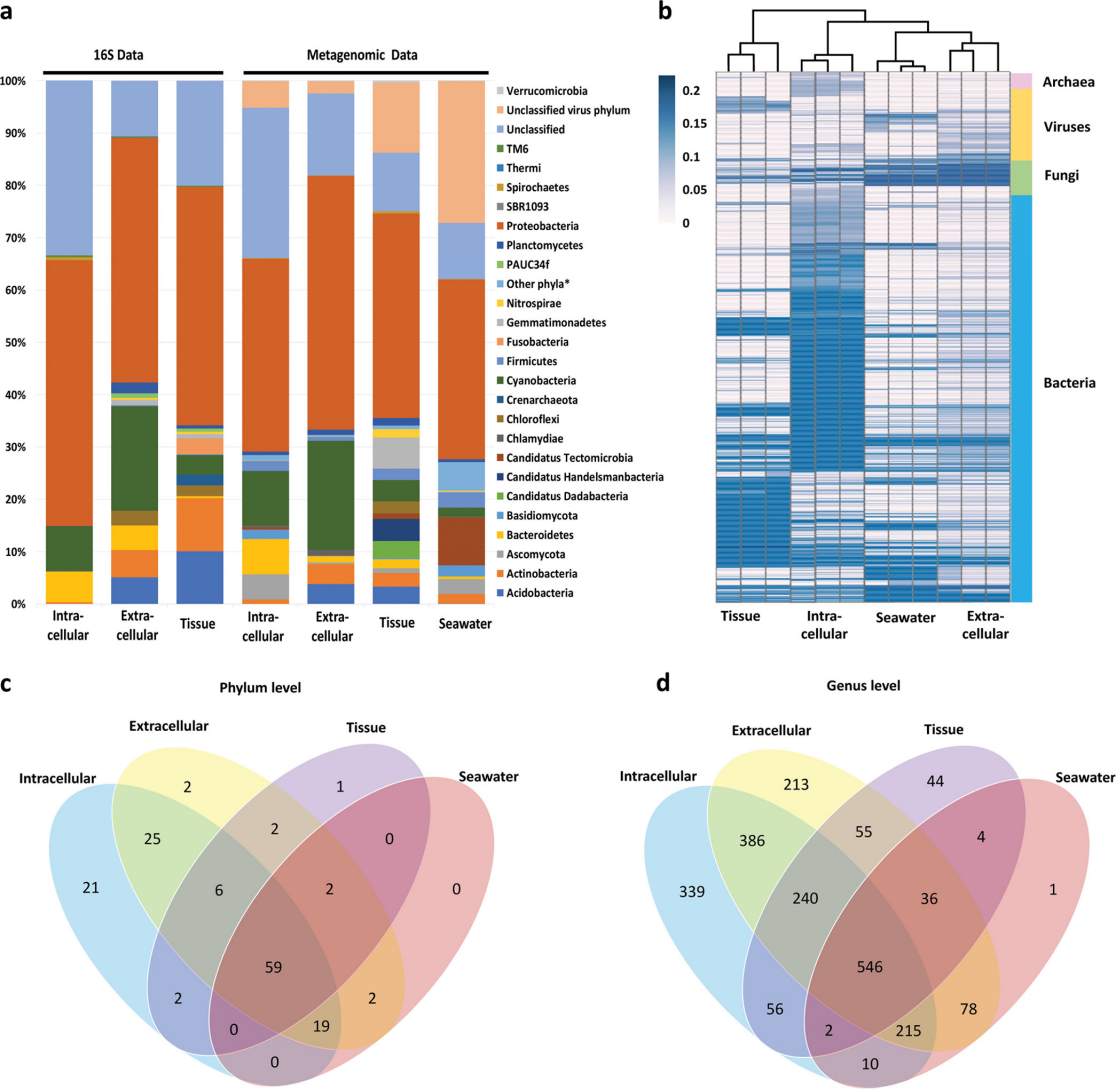
Taxonomic distribution and diversity comparisons among the four microbiomes revealed by metagenomic analysis. (a) Phylum-level taxa comparisons between 16S rRNA gene amplicon and metagenomic analysis. The metagenomic data excluded the Porifera sequences. Other phyla* includes the sum of all the rest phyla (relative abundance < 0.01% each) generated by metagenomic data. The intracellular profile by amplicon sequencing included two data sets of cell archaeocyte fraction and choanocyte fraction samples. The intracellular profile by metagenomics was based on the sequencing data of cell archaeocyte fraction and choanocyte fraction mixture. (b) Similarity among the microbiomes of the four sample types (three biological replicates) based on the relative abundance of each genus-level microbial unigenes. (c) Venn diagram for phylum-level taxa distribution among four microbiomes. (d) Venn diagram for genus-level taxa distribution among four microbiomes.

Annotations using reference sequences in the nr database assigned the unigenes to protein types and taxonomic affiliations. About 85% unigenes were assigned to prokaryotes (bacteria and archaea), 14% were viral genes, and only 1% were annotated as fungi. Metagenome sequencing significantly enlarged the microbiome coverage (141 phyla including eight fungal and one viral phyla) compared with amplicon analysis (21 phyla) (Fig. 6a), but the dominant phyla remained consistent between the two approaches, as did the relative-abundance patterns for each type of samples (Fig. 6a). Given the consistency, the dominant core microbiota in each sample type were identified based on both data sets: Acidobacteria, Actinobacteria, Cyanobacteria, Proteobacteria, and Unclassified for the tissue microbiome (89.6% and 60.2% relative sequence abundance in the amplicon and metagenomic data, respectively), as well as for the extracellular microbiome (87.7% and 92.6%). In comparison, Bacteroidetes, Cyanobacteria, Proteobacteria, Spirochaetes, and Unclassified were the core taxa for the intracellular microbiome (99.1% and 82.9%). The seawater microbiome contained members of *Ca.* Tectomicrobia, Firmicutes, Proteobacteria, Unclassified virus phylum, and Unclassified bacterial and archaeal phyla as the most dominant phyla (84.3% relative sequence abundance in the metagenomic data).

Considering all the unigenes, the profiled microbiomes of the four sample types can be clearly distinguished based on their specific composition and structure of the genus-level microbial members (Fig. 6b) that covered archaea, bacteria, fungi, and viruses. Principal coordinates analysis (PCoA) using Bray-Curtis distance also demonstrated the clearly distinguished four samples (*P* = 0.0001 by PERMANOVA) (Supplemental file 3). The extracellular and seawater samples were considerably more similar to one another than to either the intracellular or tissue samples. At the phylum level, the intracellular microbiome hosted a considerable higher number of the unique taxa than the other three communities (Fig. 6c). There were 120 sponge-derived phyla, 65 of which were shared between all the sample types. Of the 82 seawater-derived phyla, 73% were also found among the sponge samples. Consistently, down to the genus level, the greatest number of unique taxa were found in the intracellular microbiome, which included 17 archaeal genera, 267 bacterial genera, 47 fungal genera, and eight viruses genera, followed by extracellular microbiome (eight archaeal genera, 174 bacterial genera, 126 fungal genera, and five viruses genera), tissue microbiome (three archaeal genera, 37 bacterial genera, two fungal genera, and two viruses genera), and then seawater microbiome with only one bacterial genus (Fig. 6d).

### Functional specificity of the sponge intracellular microbiome uncovered by metagenome sequencing.

**FIG 7:**
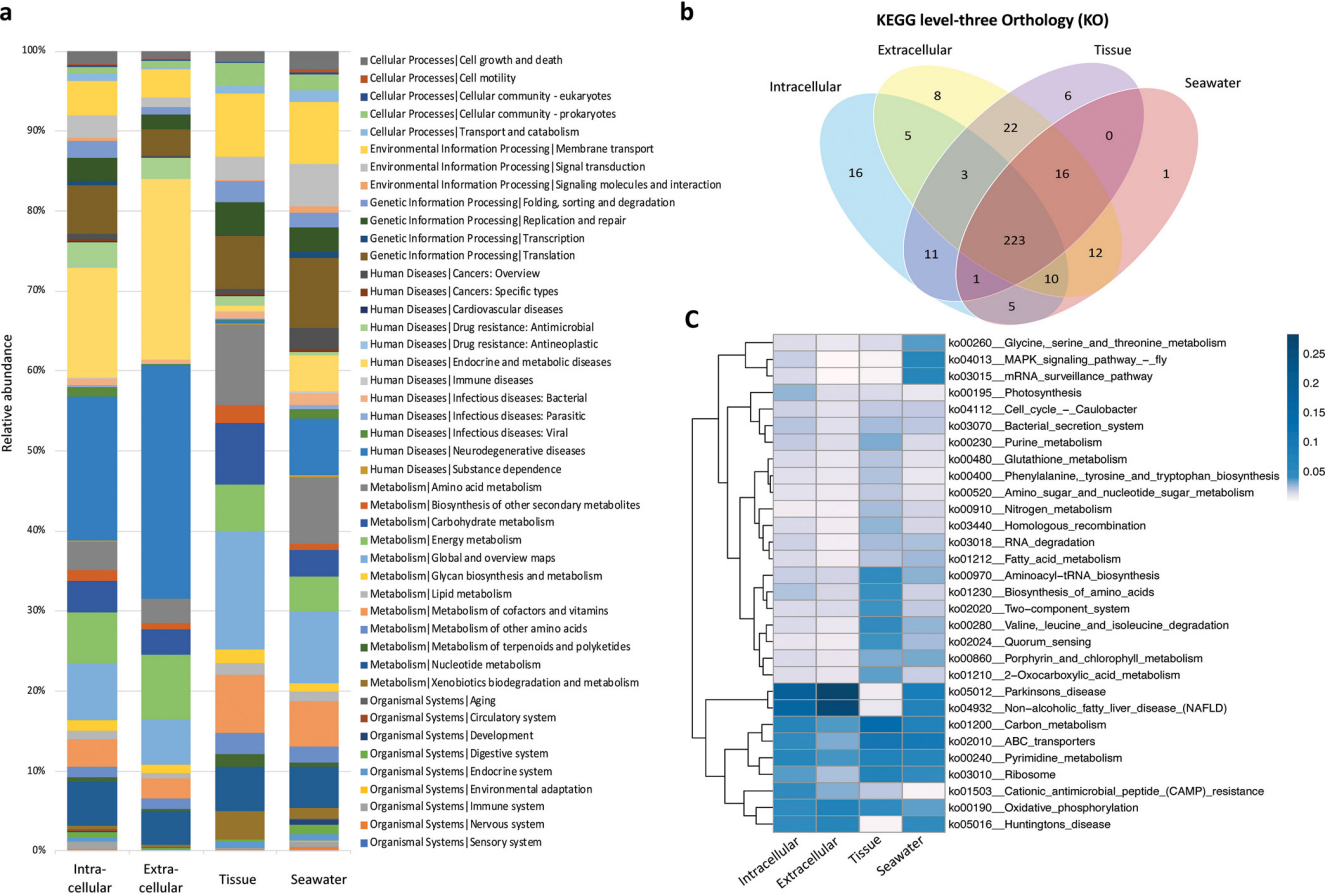
KEGG pathways comparison among the four microbiomes revealed by metagenomic sequencing. (a) Functional KEGG level-two pathway distribution. (b) Venn diagram depicting the numbers of KEGG level-three orthology (KO) in each sample. The unique 16 pathways include ko00290 (Valine, leucine and isoleucine biosynthesis), ko00250 (Alanine, aspartate and glutamate metabolism), ko00450 (Selenocompound metabolism), ko00626 (Naphthalene degradation), ko00624 (Polycyclic aromatic hydrocarbon degradation), ko00514 (Other types of O-glycan biosynthesis), ko00591 (Linoleic acid metabolism), ko00943 (Isoflavonoid biosynthesis), ko00640 (Propanoate metabolism), ko00710 (Carbon fixation in photosynthetic organisms), ko00130 (Ubiquinone and other terpenoid-quinone biosynthesis), ko05131 (Shigellosis), ko05231 (Choline metabolism in cancer), ko05110 (Vibrio cholerae infection), ko04122 (Sulfur relay system), and ko04520 (Adherens junction). (c) Heatmap depicting the distribution of the top 30 dominate level-three pathways. Clustering is based on the relative abundance of each KO.

Functional annotations were firstly obtained by a BLAST search of the clustered unigenes against the KEGG orthology (KO) database; for all microbiomes, KOs were primarily assigned to 45 KEGG level-two pathways (Fig. 7a). Within the KEGG level-three pathways, we obtained 338 sponge-derived pathways (226 shared between the groups); 99% of seawater-derived pathways were also included in this grouping. We further identified 16 unique pathways from the intracellular microbiome (Fig. 7b), a greater number than that found to be unique to the extracellular (eight), tissue (six), and seawater (one) samples. The 16 pathways belong to categories metabolism (11 pathways), human diseases (three pathways), genetic information processing (one pathway), and cellular processes (one pathway). Considering the relative abundance of each level-three pathway, it is found that the sponge tissue and seawater microbiomes contained a much more even distribution among the dominant pathways (Fig. 7c, indicating top 30 dominant KOs); in contrast, the intra- and extracellular microbiomes were highly enriched in a few KOs.

**FIG 8:**
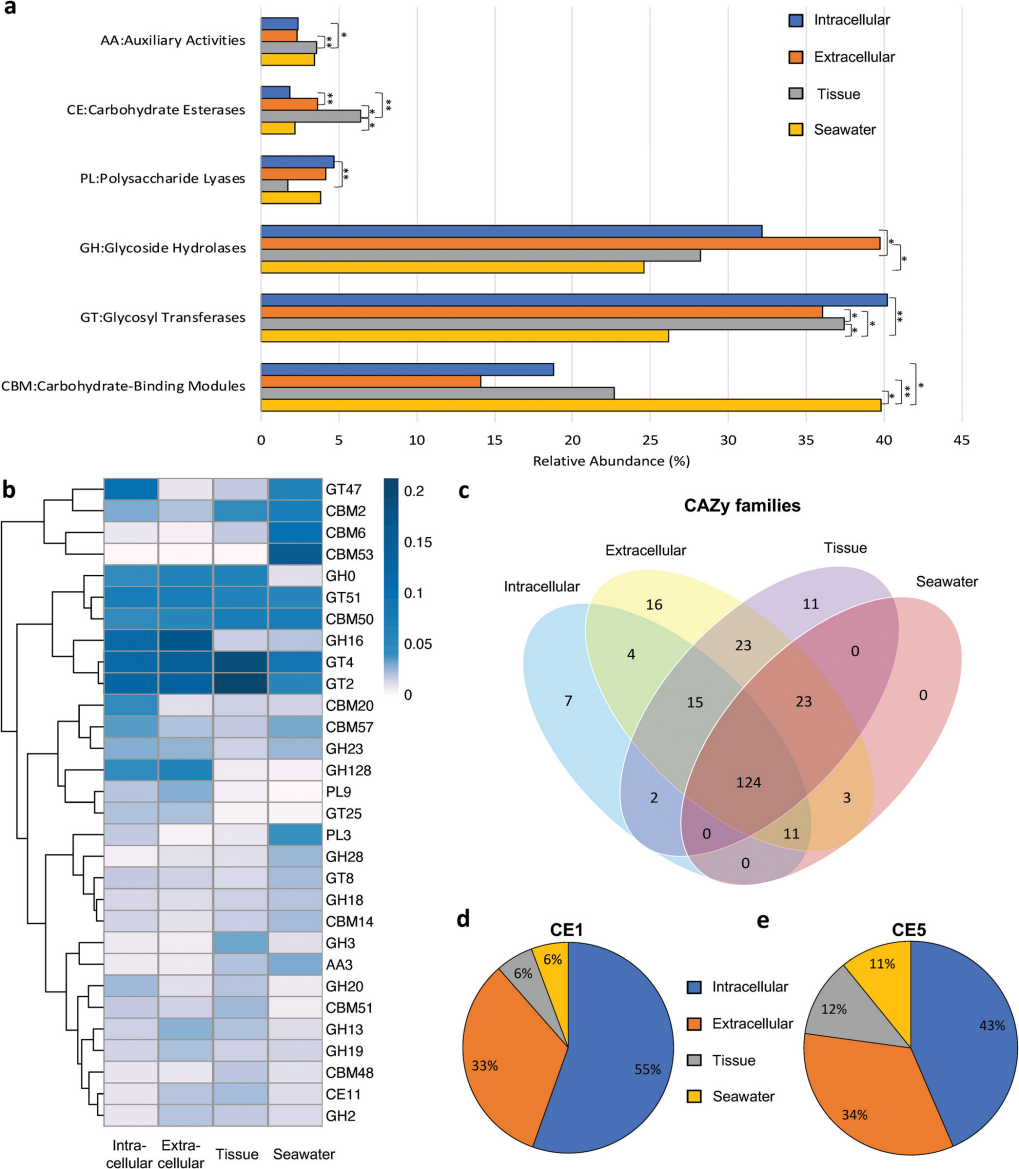
Carbohydrate-degrading enzymes (CAZy) comparison among the four microbiomes revealed by metagenomic sequencing. (a) Relative abundance of the unigenes assigned to six carbohydrate active enzyme classes. (b) Heatmap depicting the distribution of the top dominate 30 CAZy families belonging to the classes. Clustering is based on the relative abundance of each family. (c) Venn diagram depicting the numbers of CAZy families in each sample. (d) Distribution of CAZy family CE1. (e) Distribution of CAZy family CE5.

To examine differences in carbohydrate metabolism between these microbial communities, functional annotations were generated by querying the carbohydrate-active enzyme (CAZy) database, and the hierarchical annotation was analyzed based on the distribution of six enzyme classes in the different microbiomes (Fig. 8). Overall, the enzymes annotated from the metagenomes showed a high level of glycoside hydrolases (GH), glycosyl transferases (GT), and carbohydrate-binding modules (CBM) in the studied microbiomes. Pairwise comparison of the samples demonstrated the largest difference between the intracellular fraction and the tissue microbiome, with statistically significant differences in auxiliary activities (AA, *P*-value < 0.05), carbohydrate esterases (CE, *P*-value < 0.005), GH (*P*-value < 0.05), and polysaccharide lyases (PL, *P*-value < 0.005) (Fig. 8a). The most common variances between the samples were the abundances of CE and GT as they both had the greatest number of comparisons, between each other among the four microbiomes, showing statistically significant difference. The tissue sample had the largest number of significant-difference pairs against each of the other three microbiomes for different CAZy classes. Correspondingly, the family-level CAZys were also dominated by GH (family 16), GT (families 2, 4, 47, and 51), and CBM (families 2, 6, 50, and 53) (Fig. 8b). Moreover, different microbiomes can be distinguished by the enrichment of specific enzymes. There are seven, 16, and 11 unique CAZy families specific to intra-, extracellular, and tissue samples, respectively (Fig. 8c). All of the seawater related CAZys were covered by sponge-derived ones. Among the intracellular specific enzymes, we found enzymes involved in degradation (i.e., carboxyl esterase in CE10 esterases). Other typical serine hydrolases families (CE1 lipase and CE5 cutinase) were further analyzed. The results showed that they were dominant in the intracellular microbiome and had the highest abundance (55% and 43% for CE1 and CE5, respectively) compared with other three microbiomes, followed by the extracellular microbiome (33% and 34% for CE1 and CE5, respectively) (Fig. 8d and e).

### Unique functional features of the sponge intracellular microbiome.

**FIG 9:**
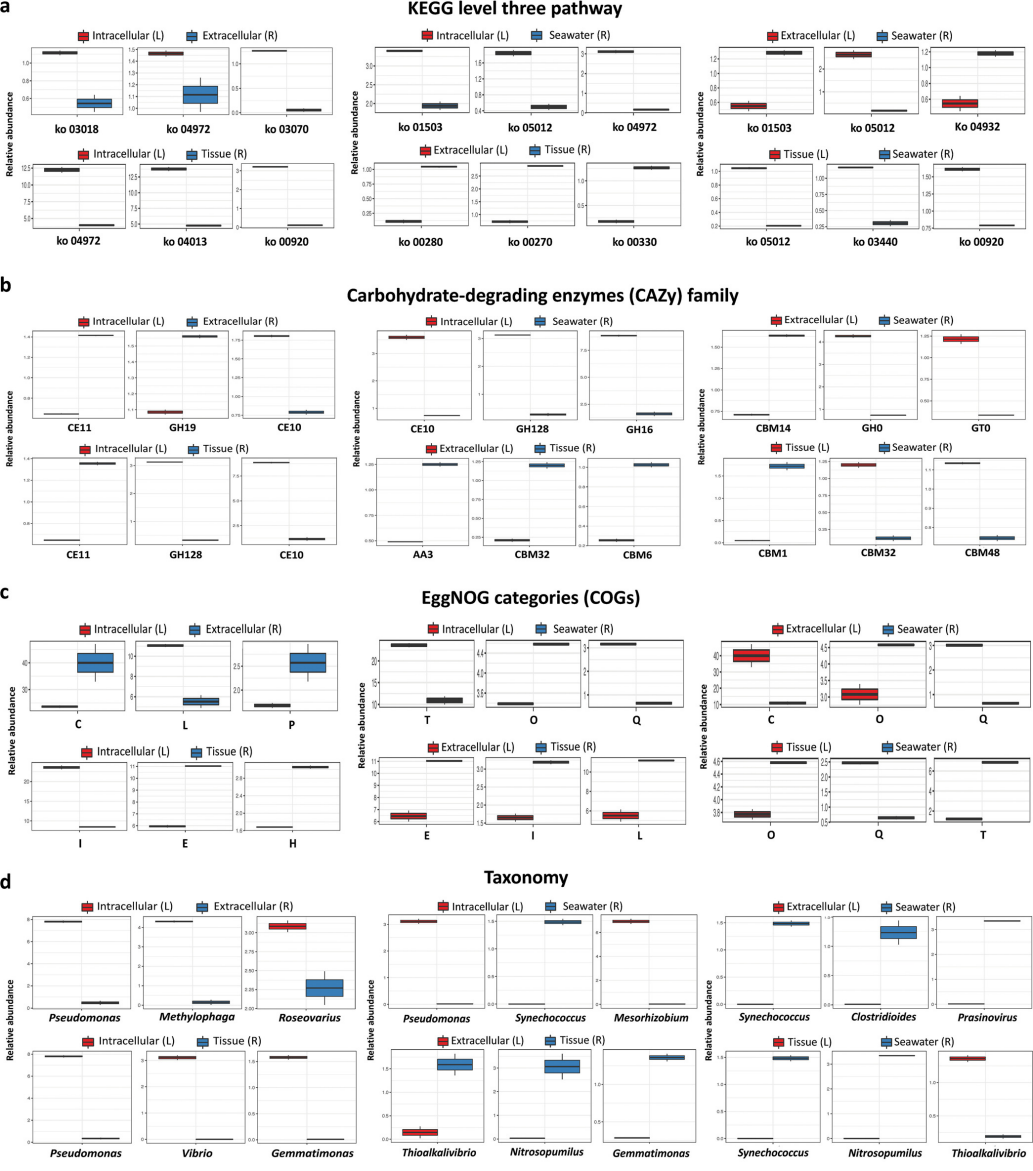
Significantly differential analysis of the functional and taxonomic features among the four microbiomes. (a) KEGG level three pathways comparison; ko 03018, RNA degradation; ko 04972, pancreatic secretion; ko 03070, bacterial secretion system; Ko 04013, MAPK signaling pathway; Ko 00920, sulfur metabolism; ko 01503, cationic antimicrobial peptide resistance; ko 05012, Parkinson’s disease; ko 00280, Valine, leucine, and isoleucine degradation; ko 00270, cysteine and methionine metabolism; ko 00330, arginine and proline metabolism; ko 01503, cationic antimicrobial peptide (CAMP) resistance; ko 04932, non-alcoholic fatty liver disease (NAFLD); ko 03440, homologous recombination. (b) Carbohydrate-degrading enzymes family level comparison. (c) EggNOG categories (COGs) comparison; C, energy production and conversion; L, replication, recombination, and repair; P, inorganic ion transport and metabolism; I, lipid transport and metabolism; E, amino acid transport and metabolism; H, coenzyme transport and metabolism; T, signal transduction mechanisms; O, posttranslational modification, protein turnover, chaperones; Q, secondary metabolites biosynthesis, transport, and catabolism. (d) Genus-level taxa comparison. The comparison is conducted between each other among the four samples. The top three results with the smallest *P*-value (< 0.05) are presented.

An analysis of the functional features and taxonomic structure of the microbiomes allowed us to better understand the specific functional roles of the sponge microbiota (Fig. 9). The significant-difference analysis indicated the three most differentially abundant KEGG pathways in each of the six pairwise comparisons (Fig. 9a). The pathway ko04972 (involved in the digestive system) was the only one consistently identified with higher abundance in intracellular microbiome compared with extracellular, tissue, and seawater microbiomes. Notably, for the analysis of CAZys, only the family CE10 (carboxyl esterase) kept a consistently higher abundance in intracellular microbiome (Fig. 9b). For the eggNOG annotated clusters of orthologous genes (COGs), the COGs belonging to groups I (lipid transport and metabolism), L (replication, recombination and repair), Q (secondary metabolites biosynthesis, transport and catabolism), and T (signal transduction mechanisms) showed higher abundances in the intracellular microbiome when compared against each of the other three microbiomes (Fig. 9c). In addition, the composition and relative abundance of each COG for the four microbial communities were distinguishable between each other (Fig. S3). To link the functions with their microbial origins, the taxa reported as lipase-producing bacteria were obtained by searching the eggNOGs database (Supplemental file 4). Among these identified bacterial genera, Pseudomonas was found to be unique to the sponge intracellular microbiome (Supplemental file 4) and showed consistently a higher abundance compared to other three microbiomes in the pairwise significant-different analysis (Fig. 9d).

## DISCUSSION

Our study validated that the strategy of directly sequencing different cell fractions of sponge tissue, in particular the sponge stem cell population, could offer a more comprehensive profile of sponge microbiomes. Using the sponge *Euryspongia arenaria* as a model, we found the endosymbiotic microbiome hosted unique taxa from the phylum to genus level compared to the extracellular, tissue, and surrounding seawater microbiomes. The functional specificity of endosymbiotic microbiota was demonstrated by their unique pathways and enzyme families.

To profile the sponge endosymbiotic microbiome using community DNA extraction and next generation sequencing, it is critical to obtain a pure sponge cell fraction. Therefore, for different sponge species the most challenging step is to develop a purification strategy specific to the composition of that sponge. Typically, differential centrifugation is a well-developed method to separate the cells based on their different relative densities (68–70). Density gradient centrifugation, usually with Ficoll as the medium, has also been commonly used in the fine-scale sponge cell separation (71–80). It was noted in our study that the cells of *E. arenaria* were highly sensitive to the dissociation conditions and centrifugation (Fig. S1). A cell purification protocol (Fig. S4a) was developed and validated after optimization tailored to the properties of different *E. arenaria* cell types. Cell aggregation (81–84), a unique characteristic of sponge cells, was additionally employed as an efficient approach to further enrich cells by excluding damaged sponge cells. The formation of *E. arenaria* primmorphs thereby selected for healthy sponge cells and removed the contaminants or heterogenous cells, such as dead and broken sponge cells (Fig. S4b). This combined *in vitro* cultivation-differential centrifugation strategy greatly improved the cell purification efficiency (Fig. 1a to i) and produced two target cell fractions (Fig. 1j to m). The purification efficiency was further validated by the statistically consistent Raman spectra of individual cells within each fraction (Fig. 3), demonstrating their chemical homogeneity. TEM examination eventually distinguished and identified the cell types in our study (Fig. 2a and b) and revealed the presence of endosymbiotic microbes in both archaeocytes (Fig. 2c) and choanocytes (Fig. 2d). Matching the reported morphological features of archaeocytes (85, 86), we also found that the cells enriched in fraction I are motile and relatively large and contain a large nucleus with a single large nucleolus. For the cells in fraction II, we identified choanocytes due to their most critical morphology feature, i.e., flagella (65, 86).

Unlike the traditional sponge microbiome investigations, in which DNA isolated directly from sponge tissues is sequenced, the strategy here provided a new insight into the completeness of the sponge microbiome. The comprehensive data obtained by investigating preseparated fractions of sponges greatly impacted the observed coverage of the microbial profile (Fig. 4e and Fig. 6b). The profile generated by bulk sequencing of the sponge tissue sample missed out 14% (three out of 21) of microbial phyla and 62.8% (91 out of 145) of the identifiable genera (Fig. 4c and d; Table S1b and c). This is qualitatively consistent with similar studies in which the enrichment of the microbial fraction in a metagenome has also been reported to improve the resolution of microbiome characterization (44, 56–58, 60, 87). However, these investigations did not focus on the comparison between the revealed microbiomes pre- and post-enrichment of the microbial fraction. Only one study on two sponges (*Cymbastela concentrica* and *Scopalina* sp.) (59) found that the microbial OTUs obtained by a sequencing of whole sponge tissue had only a small overlap with those revealed from the bacterial pellet, which is consistent with the finding in our study that parts of the sponge microbiome could be hidden depending on the sample processing strategy (Fig. 4d and Fig. 6d). Based on these findings, it seems likely that the substantial differences were due to a sample preparation approach that allows for more efficient recovery of DNA from the microbial community. When the generated microbiomes highly depend on DNA quality, a more focused sequencing from bacteria-enriched fractions (intra- and extracellular) can therefore offer more comprehensive sequencing results. Our study paves the way toward approaching a complete microbiome for any other marine sponges.

Most sponge symbiotic microorganisms inhabit the mesohyl tissue, which constitutes most of the sponge body and is formed of an extracellular matrix populated by sponge cells (88). Only through TEM have researchers gradually become aware of the presence of intracellular bacterial symbionts in these tissues (86, 89–97). Consumption of symbiotic microorganisms has been proposed as a possible food source for sponges (98, 99), and indeed phagocytosis and subsequent intracellular digestion of bacteria are the presumed mechanisms of nutrient transfer between a carnivorous sponge *Cladorhiza* and its methanotrophic symbionts (100, 101). Calcification was reported to be mediated by endosymbiotic bacteria (calcibacteria) within archeocyte-like cells of sponges in genus *Hemimycale* (61–63). A recent study sorted sponge homogenate based on particle size and localized the production of defense chemicals to intracellular symbionts of *Haliclona* sponges (64).

In our study, we focused on two sponge cell types and conducted a tailored cell sorting and purification so as to produce a detailed profile of the microbiome both intra- and extracellularly using our recently developed efficient multi-primer amplicon sequencing (102) and shotgun metagenomics. The different types of enriched sponge cells hosted specific microbial taxa, extending from genus to phylum, (Fig. 4) that were distinct from the ones of the extracellular and tissue samples. Notably, the community comparison indicated a high similarity between two cell fractions (Fig. 4a and e), which also guided us to pool them together for the shotgun metagenomic analysis. The microbiome structural specificity was further explained by additionally comparing it with the seawater sample (Fig. 6). The unique taxa specific to the intracellular microbiome were identified with significantly increased taxa number by using metagenomes. Interestingly, Archaea were found almost exclusively in the intracellular fractions. In contrast, their virus abundances were much less than the other three microbiomes (Fig. 6b).

As is common with such studies, a substantial percentage of the sequences we obtained by amplicon or metagenome sequencing could not be classified taxonomically. In our study, only 34% of the 16S rRNA gene amplicons could be identified in the archaeocytes-enriched fraction, 33% in the choanocytes-enriched fraction, 20% in tissue, and 11% in extracellular bacteria fraction (Fig. 4c). Although amplicon sequencing provides less capacity to explore untapped microbial taxa from a complex microbiome, the advantage of the primer based 16S rRNA gene sequences is that they can be effectively BLASTed against comprehensive databases such as Greengenes to identify the microbial taxa. Each of the multiple representative sequences assigned to the same genus or family (Fig. 5) were re-classified to a deeper taxonomic level for a better taxonomic resolution (Data sets S2). The inferred taxa include the species belonging to Alpha-, Gamma-, and Epsilon-proteobacteria, as well as Bacteroidetes and Cyanobacteria, which could represent microbial resources for gene and metabolite discovery.

Sponge symbionts have been shown to be the actual producers of the diverse natural products with a range of important bioactivities (64, 103–106). Among these natural products producers, cultivated bacteria represent only a minute fraction and the uncultured majority are generally perceived as a large, untapped resource of new drug candidates. Sponges belonging to the genus *Euryspongia* have been shown to contain abundant secondary metabolites with various activities (15–24). However, there are no comprehensive metagenomic annotations for the microbiomes of the sponges *Euryspongia* to allow for a correlation analysis between the functions and their potential microbial origins. The presence of functional features specific to the intra- or extracellular microbiomes was demonstrated by KEGG pathway distribution (Fig. 7); in particular, the highest number of unique level-three pathways was identified in the intracellular microbiome (Fig. 7b). The functional specificity of the intracellular endosymbionts can be further distinguished using their carbohydrate-active enzymes (CAZys) profile (Fig. 8a to c). Moreover, the statistically differential comparison guided us to identify an KEGG pathway (ko 04972) and a CAZy family (CE10) specific to the endosymbiotic microbiome (Fig. 9a and b). The enrichment of the pathway ko04972, designated by KEGG as the pancreatic secretion pathway involved in digestive system, may be related to a high abundance of muscarinic acetylcholine receptor and cholecystokinin receptors (107–109), likely acquired by horizontal gene transfer and possibly required for the maintenance of an endosymbiotic lifestyle (110–112). Comparing the involved enzymes in the pathway with the ones belonging to family CE10, we found one shared entry-triacylglycerol lipase (EC 3.1.1.3) that was applied as polyethylene terephthalate hydrolase (113). In addition, the eggNOGs v5.0.0 database (114) supports the correlation analysis to link the annotated functional COGs and enzymes with their microbial origins, which allowed us to understand which microbial taxon carries which specific function. By doing so, we identified 40 microbial species containing homologs of triacylglycerol lipase (EC 3.1.1.3) enzymes that matched with the unique taxa in intracellular microbiome (Supplemental file 4). Consistently, the predicted COGs (i.e., functional categories L, T, and I) for this lipase family (EC 3.1.1.3) were also found to be specific to the intracellular microbiome with relative high abundance (Fig. 9c). Moreover, among the matched 40 microbial taxa, Pseudomonas members were identified to be specific to the intracellular microbiome by statistical differential analysis at the microbial genus-level comparison (Fig. 9d). These findings provide a valuable guidance to conduct studies on the functional gene repertoire of intracellular microbiomes in diverse sponges.

In conclusion, sequencing pure sponge cell fractions by combining both amplicon and metagenomic data provided a comprehensive analysis of the *E. arenaria* microbiome and revealed for the first time the structural and functional specificity of the intracellular endosymbiotic microbiome compared to its extracellular, tissue, and seawater microbiomes. The sponge cell purification and extracellular bacteria enrichment could be considered as an approach for profiling a complete sponge microbiome, in particular for uncovering the low abundant and likely missed microbiota when sequencing the sponge bulk tissues. Metagenome-based annotations of various features revealed potential functions correlated to the unique intracellular microbiota. One promising finding on metagenome-annotated serine hydrolases families enriched within intracellular microbiome highlighted the sponge endosymbiotic taxa as an understudied source for novel functional bacteria resources mining. Additional downstream analyses, such as conducting a whole-genome assembly from metagenomes to identify gene clusters for natural products or applying single-cell sequencing to directly reveal the sponge endosymbionts, are still necessary to further our understanding and utilization of sponge intracellular endosymbionts.

## MATERIALS AND METHODS

### Sponge sample collection and identification.

Sponge specimens (*n* ≥ 3 in each of the two collections) were collected via scuba diving at depths of 4 to 15 meters at Rapid Bay, Adelaide, South Australia (35°31'16.6"S, 138°11'07.5"E) in January of 2018. The healthy sponge specimens were soaked in flowing natural seawater to clean the contaminations. Preparation for histological sections and spicule preparations followed the methods in “Sponguide” (115). The classification refers to the study for Demosponge classification (116). The specimens were identified as *Euryspongia arenaria* (Queensland Museum Registration No. G301355) (Class: Demospongiae; Order: Dictyoceratida; Family: Dysideidae) using morphological features (117). Seawater samples were collected in parallel with sponge sampling using 50 mL tubes (*n* = 3) and kept in −80°C freezer for further analysis.

### Sponge cells and extracellular bacteria enrichment.

Three sponge specimens were soaked in sterile calcium/magnesium-free artificial seawater (CMFASW) for to remove natural seawater. The sponge tissues were then fixed using 2.5% glutaraldehyde in CMFASW. The cell dissociation protocol (Fig. S4a) was developed based on the study (79). The cell purification was conducted following the protocol shown in Figure S4b (79). Continuous Ficoll-CMFASW gradients with densities of 10%, 20%, 30%, and 40% (wt/vol %) were applied to separate the sponge cells. One cell fraction was collected between 20% and 30% Ficoll-CMFASW gradients; the other fraction was collected between 10% and 20% gradients. The pellets from high-speed centrifugation were kept for extracellular bacterial community analysis.

### Sponge cell type characterization and endosymbiont identification.

The morphology and structure of sponge cells in the two purified fractions were characterized by TEM based on a protocol (118) with optimizations. Briefly, ultrathin sections were obtained with the Ultramicrotome Leica EM UC7 and stained first with 2% uranyl acetate for 30 min and then with lead citrate for 15 min at room temperature. Observations were conducted using a FEI Tecnai G2 Spirit TEM with Olympus-SIS Veleta CCD camera. Analysis was conducted via an EDS system comprising an Apollo XLT SDD running EDAX’s TEAM software.

### Raman microspectroscopy.

*In situ* measurement of chemical composition within each single sponge cell was recorded with a WITec Alpha 300R Raman microscope (WITec Instrument Corp., Germany) equipped with CCD detector (119, 120). The confocal Raman spectra were generated following the instrument operating procedure. 10 μL suspension of purified sponge cell fraction was dropped onto middle of a glass slide and then covered by a cover slip. Five slides (five subfractions) were prepared for each cell fraction. Microscope was adjusted manually for focusing before turning on the Raman laser. Five investigation areas were selected for each slide for data collection: (i) top left corner; (ii) top right corner; (iii) middle; (iv) bottom left corner; and (v) bottom right corner. Four sponge cells in each area were selected randomly to profile single Raman spectra using 532 nm laser after manual validation of the laser intensity.

### DNA extraction.

The DNA extraction protocol utilized for sponge tissue is the Cetyltrimethylammonium bromide (CTAB)-based method (39, 121) with the modifications described in Yang et al. (122). For purified sponge cell fractions and enriched bacteria fraction, the extraction was conducted by an extraction kit Quick-DNA/RNA Microprep Plus Kit (ZYMO Research, USA, Cat. No. D7005). Three biological replicates for each of the five types of the samples (sponge tissue, two cell fractions, extracellular bacteria sample, and seawater sample) were applied. Purity and quantity of DNA were determined with a NanoDrop ND-1000 Spectrophotometer (Thermo Fisher Scientific, Wilmington, DE, USA). Each sample was extracted in triplicate. The qualified DNA samples (A260/280: 1.8-2.0; Conc. > 100 ng/μL) extracted from three replicates for each sample type were selected and kept at −20°C for subsequent PCRs and sequencing.

### Illumina MiSeq amplicon library and sequencing.

A multiprimer based amplicon sequencing approach, which was developed and validated in our previous studies (102, 123), was utilized. Three primer sets (28F-519R, 518F-926R, and 803F-1392R) were employed for 16S rRNA gene region V1 to V8. For each primer set, negative controls and multiple amplifications (*n* > 3) were applied for each of the three qualified DNA samples extracted from different sample types. Each pooled amplicon was sequenced multiple times (*n* ≥ 3) from both ends of paired-end library preparations (2 × 300 bp) using sequencing kit version 3.0. The raw data was processed by base-calling using Illumina Genome Analyzer Pipeline software (GAPipeline version 1.4.0.). The DNA standard provided by ZymoBIOMIC was employed as the quality control (both cellular standard and DNA standard) for each sequencing run (Fig. S5).

### 16S rRNA gene sequencing data processing and analysis.

The demultiplexing and quality filter (at Phred ≥ Q20) was processed by script split_libraries.py in QIIME pipeline (version 1.9.1) (124). The primer sequences and the poor quality sequences were trimmed by the software Trimmomatic (version 0.35) (125). Chimeric sequences were removed by the USEARCH tool (126). A minimum of 99,864 filtered sequence reads per primer set was collected for each biological replicate of same sample type. The sequencing reads generated by three primer sets were jointly applied based on the previous methodology validation (102).

Closed-reference picking was selected in our study (124). Reads were assigned to OTUs based on their best hit to Greengenes database (version: gg_13_8_otus/rep_set/97_otus.fasta) (127) at greater than or equal to 97% sequence identity (128). Greengenes was selected as the reference gene database due to its intermediate number of the sequences and sufficient taxa coverage at each rank from phylum to genus compared to SILVA and RDP (129, 130). While SILVA is a comprehensive database, the risk of overestimating the diversity is high due to the highest number of false-positives (130). In addition, the multi-primer approach applied in this study has demonstrated powerful performance on increasing the revealed microbial taxa coverage and the classification accuracy (102, 123). Hence, our analysis was based on Greengenes database. The uclust (126) consensus taxonomy classifier was applied to assign taxonomy to each OTU. The taxonomy metadata was further conducted by ClustVis to visualize the microbial community heatmap (67) with the implementation of R package of “pheatmap.” In the heatmap, both rows and columns are clustered using “correlation” distance measurement and “complete linkage” clustering method. The rows are centered, and the unit scaling is applied. Unique-sequence abundance heatmap was built directly from the abundance of unique amplicon sequences inferred from raw sequencing data. To select sequence variants, DADA2 plugin (131) was implemented in QIIME2 pipeline (https://qiime2.org) to generate the exact “sequence variants” based on statistical error correction model. The involved unique sequences were exported as FASTA format data to conduct BLAST search for subspecies identification.

### Illumina HiSeq metagenomic library preparation and sequencing.

Two purified sponge cell fractions I and II were pooled for metagenomic sequencing. Library preparations were constructed following the manufacturer’s protocol (VAHTS Universal DNA Library Prep Kit for Illumina). Replicates were applied. The PCR products were cleaned up using VAHTSTM DNA Clean Beads, validated using an Agilent 2100 Bioanalyzer (Agilent Technologies, Palo Alto, CA, USA), and quantified by Qubit 3.0 Fluorometer (Invitrogen, Carlsbad, CA, USA). Then libraries with different indices were multiplexed and loaded on an Illumina HiSeq instrument according to manufacturer’s instructions (Illumina, San Diego, CA, USA). Sequencing was carried out using a 2 × 150 paired-end configuration on Illumina HiSeq instrument.

### Metagenomic data processing and analysis.

The raw reads from metagenome sequencing were processed by cutadapt (v2.0) (132) to remove adaptor sequences and low-quality reads. The reads were then aligned to the host genome using BWA (v0.7.13) (133, 134) to remove host contamination. The quality optimized clean reads were then processed by MEGAHIT (v1.1.3) (135) for *de novo* assembly. Different K-mers (39, 59, 79, 119) were tested, and the value was selected based on the largest N50 of the assembled scaffolds. Contigs (continuous sequences within scaffolds) with more than 200 bp were retained in the final assembly for gene prediction. The metagenes were predicted using Prodigal (v3.02) (136). CD-HIT (v2.21) (137) was applied to reduce sequence redundancy and cluster the metagenes with the parameters of minimum identity 0.95 and coverage over 0.9. Approximate 2.4 million non-redundant metagenes (unigenes) were obtained.

To generate the taxonomic information of the unigenes, the sequences were blasted against the NCBI microbial nr (non-redundant protein) database using DIAMOND (v0.8.15.77) (138) with an *E* value < 1e^−5^. Based on the lowest common ancestor algorithm in MEGAN (v6.4.4) (139), the taxonomic identities of each unigene were assigned. The sequences were further blasted against the KEGG database (for KOs and pathways), CAZy database (for carbohydrate-active enzymes), and eggNOG database (for COGs and their host organisms) using DIAMOND with E value < 1e^−5^. For the taxonomic and functional abundance profiling, the reads belonging to different samples were mapped separately against all the unigenes using SOAPAligner (140) with the default setting.

To determine the similarity or difference of taxonomic components between different samples, clustered heatmap was constructed based on the relative abundance of each unigene at the genus level. The dendrogram was generated by R (pheatmap package) using *complete* method based on *bray* distance. T-test was employed to indicate the significant-difference pairs for CAZy abundance annotation. The statistical difference comparison among the samples were conducted by Metastats (141) to identify the taxonomic and functional features.

### Data availability.

Raw 16S rRNA gene sequences, the metagenomic sequencing reads and assembly have been uploaded to the NCBI Sequence Read Archive (SRA) data depository, with the BioProject accession number PRJNA699379.
