# Supplementary material for: Marine Sponge Endosymbionts: Structural and Functional Specificity of the Microbiome within Euryspongia arenaria Cells
Source: Microbiol Spectr. 2022 May 2;10(3):e02296-21. doi: 10.1128/spectrum.02296-21 (PMC9241883; doi:10.1128/spectrum.02296-21)
Supplement: SUPPLEMENTAL FILE 5 — Supplemental material. Download spectrum.02296-21-s005.pdf, PDF file, 2.7 MB [file spectrum.02296-21-s005.pdf]

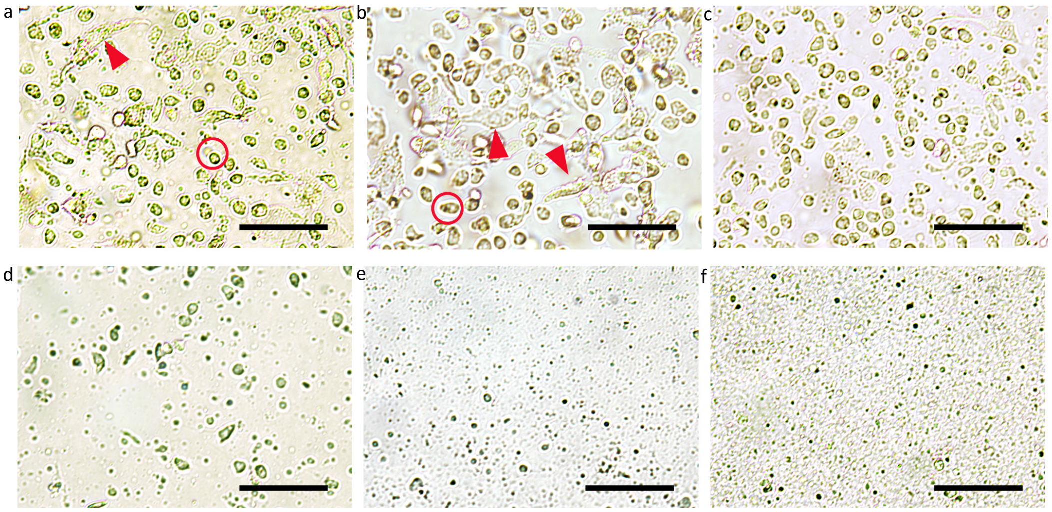

Fig. S1. Microscopy images of sponge cells and extracellular bacteria. a. Sponge cells mixture; b. 50 × g centrifugation pellet; c. 100 × g centrifugation pellet; d. 400 × g centrifugation pellet; e. 2,000 × g centrifugation pellet; 16,000 × g centrifugation pellet. Scale bar = 50 μm. Triangle, archaeocytes; Circle, choanocytes.

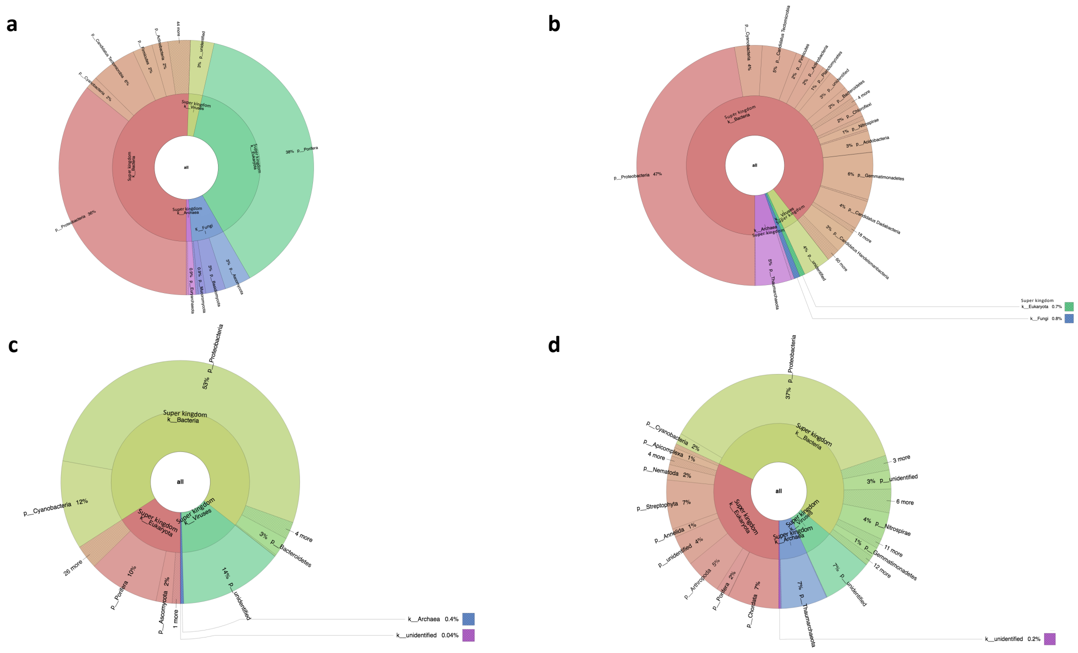

Fig. S2. Metagenomic data throughput at the kingdom level. a. Intracellular microbiome; b. Extracellular microbiome; c. Tissue microbiome; d. Seawater microbiome.

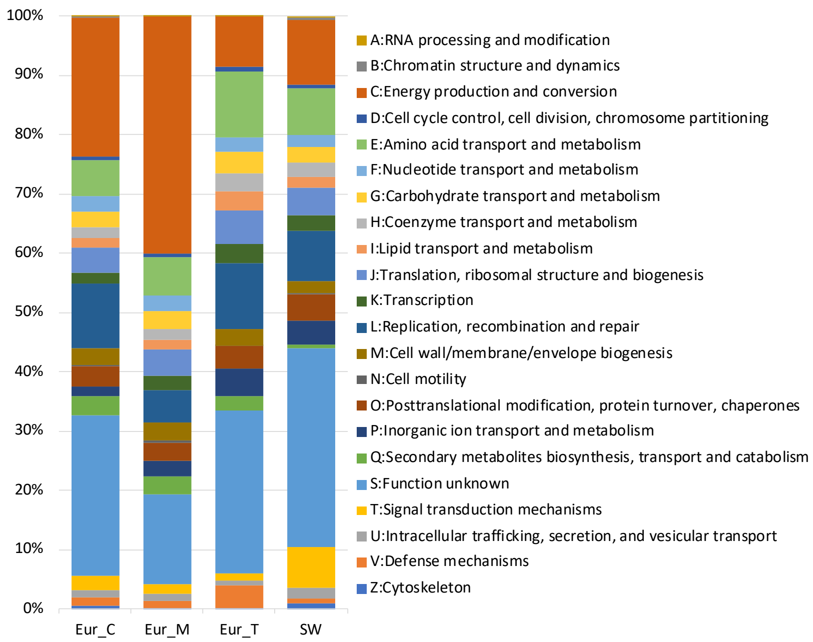

Fig. S3. Clusters of Orthologous Groups (COGs) distribution based on eggNOG database. Eur_C refers to intracellular microbiome, Eur_M refers to extracellular microbiome, Eur_T refers to tissue microbiome, and SW refers to seawater microbiome.

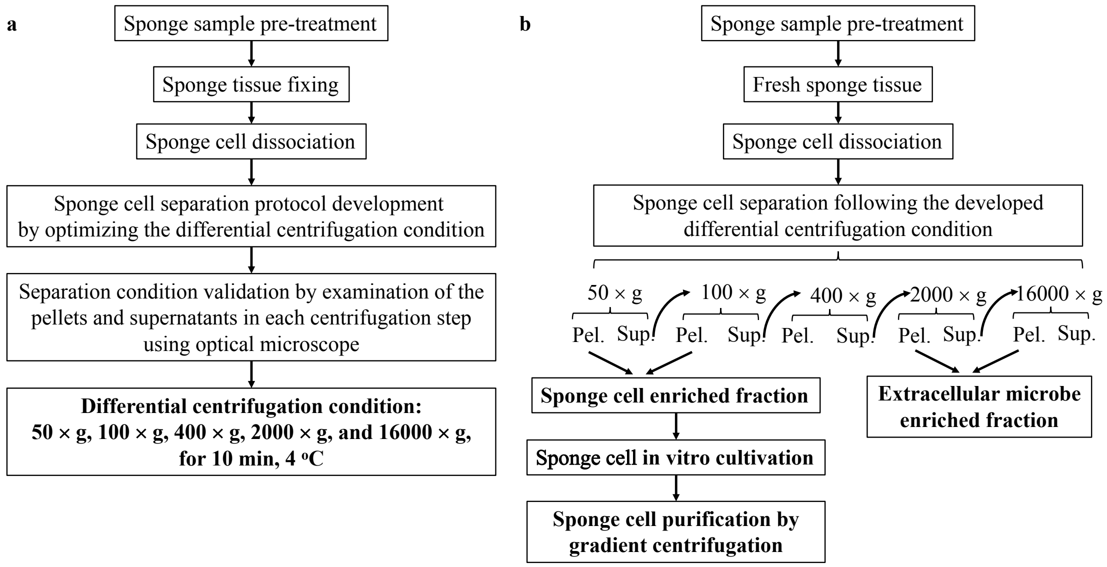

Fig. S4. Sponge cell separation and purification protocol. a. Flowchart of sponge cell separation approach development; b. Validated protocol of sponge cell purification.

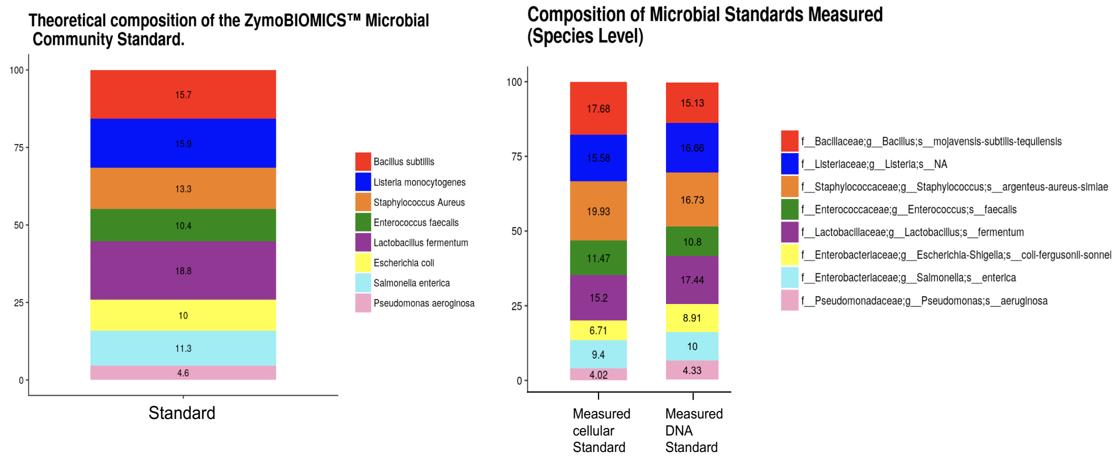

Fig. S5. Theoretical microbial composition and measured composition of the ZymoBIOMICS™ Microbial Community Standard. Cellular Standard: bacterial cultures were mixed so that communities were comprised of equal numbers of cells; DNA Standard: DNA was extracted from pure bacterial cultures and then mixed so that communities were comprised of equal amounts of DNA.

Table S1a. Summary of 16S rRNA gene based amplicon sequencing reads during data processing.

Sample type	Biological replicate	Raw seqs (R1+R2)	Trimmed seqs (R1+R2)	Inferred	Chimera seqs	Chimera free seqs	Unique seqs	Seqs (after size filtration)	Final unique seqs
Sponge tissue	1	225820	204322	99898	25	99873	316	99864	315
	2	225900	204400	99922	30	99880	315	99869	314
	3	226005	204822	99988	30	99913	316	99900	315
Sponge cell fraction I	1	544462	508854	244457	1686	242771	349	242592	324
	2	544502	508900	244501	1699	242811	355	242610	320
	3	544382	508444	244410	1629	242714	328	242574	323
Sponge cell fraction II	1	601916	558808	269461	1438	268023	356	267760	313
	2	601800	558414	269421	1405	268000	333	267555	310
	3	601696	558000	269399	1400	267900	313	267300	311
Bacteria fraction	1	543080	508086	247323	2029	245294	337	245010	293
	2	542899	507777	247100	2001	245000	319	245000	292
	3	543201	508199	247503	2059	245414	356	245070	291

Table S1b. Microbial phyla distribution with relative abundance among the communities of purified sponge cell fraction I, II, enriched extracellular bacterial fraction, and tissue sample revealed by amplicon sequencing.

#OTU ID	Tissue	Cell fraction I	Cell fraction II	Bacteria fraction
Acidobacteria	0.10084563	0	0	0.051205447
Actinobacteria	0.10121212	0.004080185	0.003170849	0.051976565
Bacteroidetes	0.003907072	0.057067814	0.060244429	0.046775734
Chlamydiae	0	0	0.001233162	0
Chloroflexi	0.02065388	0	0	0.028127875
Crenarchaeota	0.02015623	0	0	0.000098989
Cyanobacteria	0.03787856	0.095167253	0.075152559	0.199913381
Firmicutes	0.00159342	0	0	0.002034512
Fusobacteria	0.03005492	0	0	0.000086324
Gemmatimonadetes	0.00832041	0	0	0.010000532
Nitrospirae	0.00432156	0	0	0.003984512
PAUC34f	0.00612312	0	0	0.008312391
Planctomycetes	0.00649175	0.001160214	0.000651369	0.020785649
Proteobacteria	0.455490438	0.499357774	0.516588498	0.467899688
SBR1093	9.99092E-05	0.000195848	0.000195411	9.99092E-05
Thermi	8.42346E-05	0	0	7.84532E-05
TM6	0.001866262	0.003337997	0.003627615	0.002166262
Unassigned	0.200795906	0.33517885	0.332203593	0.106301466
Verrucomicrobia	0.000104578	0	0	0.000152311
Spirochaetes	0	0.004261723	0.006732512	0
Candidatus Tectomicrobia	0	0.000192342	0.000200003	0

Table S1c. Known microbial genera distribution among the communities of purified sponge cell fraction I, II, enriched extracellular bacterial fraction, and tissue sample revealed by amplicon sequencing.

	Cell fraction I	Cell fraction II	Tissue	Bacteria fraction
k__Archaea;p__Crenarchaeota;c__Thaumarchaeota;o__Cenarchaeales;f__Cenarchaeaceae;g__Nitrosopumilus	0	0	0.000396578	4.65796E-05
k__Archaea;p__Crenarchaeota;c__Thaumarchaeota;o__Nitrososphaerales;f__Nitrososphaeraceae;g__Candidatus Nitrososphaera	0	0	5.67E-05	0
k__Bacteria;p__Actinobacteria;c__Actinobacteria;o__Actinomycetales;f__Corynebacteriaceae;g__Corynebacterium	0	0	0	0.000129316
k__Bacteria;p__Actinobacteria;c__Actinobacteria;o__Actinomycetales;f__Propionibacteriaceae;g__Propionibacterium	0	0	0	0.000258632
k__Bacteria;p__Actinobacteria;c__Actinobacteria;o__PeM15;f__bacterium rJ7;g__Bacterium	0.002040092	0.001585425	0	0
k__Bacteria;p__Bacteroidetes;c__[Rhodothermi];o__[Rhodothermales];f__[Balneolaceae];g__Balneola	0	0	5.67E-05	0
k__Bacteria;p__Bacteroidetes;c__Cytophagia;o__Cytophagales;f__[Amoebophilaceae];g__Candidatus Cardinium	0	0	8.50E-05	0.000258632
k__Bacteria;p__Bacteroidetes;c__Cytophagia;o__Cytophagales;f__Flammeovirgaceae;g__JTB248	0	0	0	0.000258632
k__Bacteria;p__Bacteroidetes;c__Cytophagia;o__Cytophagales;f__Flammeovirgaceae;g__Persicobacter	0	0	0.00028327	0
k__Bacteria;p__Bacteroidetes;c__Cytophagia;o__Cytophagales;f__Flammeovirgaceae;g__Roseivirga	0	0	8.50E-05	0
k__Bacteria;p__Bacteroidetes;c__Flavobacteriia;o__Flavobacteriales;f__Cryomorphaceae;g__Fluviicola	0	0	8.50E-05	0
k__Bacteria;p__Bacteroidetes;c__Flavobacteriia;o__Flavobacteriales;f__Flavobacteriaceae;g__Algibacter	0.000165273	0.000207503	0	0
k__Bacteria;p__Bacteroidetes;c__Flavobacteriia;o__Flavobacteriales;f__Flavobacteriaceae;g__Aureimarina	0.000833779	0.001096886	0	0
k__Bacteria;p__Bacteroidetes;c__Flavobacteriia;o__Flavobacteriales;f__Flavobacteriaceae;g__Capnocytophaga	0	0	0	0.000387948
k__Bacteria;p__Bacteroidetes;c__Flavobacteriia;o__Flavobacteriales;f__Flavobacteriaceae;g__Flaviramulus	0.000147406	0.000282959	0	0

k__Bacteria;p__Bacteroidetes;c__Flavobacteriia;o__Flavobacteriales;f__Flavobacteriaceae;g__Flavobacterium	0	0	0.000945442	0
k__Bacteria;p__Bacteroidetes;c__Flavobacteriia;o__Flavobacteriales;f__Flavobacteriaceae;g__Gaetbulibacter	0.00111671	0.001344914	0	0
k__Bacteria;p__Bacteroidetes;c__Flavobacteriia;o__Flavobacteriales;f__Flavobacteriaceae;g__Ichthyenterobacterium	0.000557795	0	0	0
k__Bacteria;p__Bacteroidetes;c__Flavobacteriia;o__Flavobacteriales;f__Flavobacteriaceae;g__Ichthyenterobacterium-Mesoflavibacter	0.002164245	0.002382149	0	0
k__Bacteria;p__Bacteroidetes;c__Flavobacteriia;o__Flavobacteriales;f__Flavobacteriaceae;g__Lacinutrix	0.000456152	0.000502485	0	0
k__Bacteria;p__Bacteroidetes;c__Flavobacteriia;o__Flavobacteriales;f__Flavobacteriaceae;g__Leeuwenhoekiella	0.000114038	0.000136477	0	0
k__Bacteria;p__Bacteroidetes;c__Flavobacteriia;o__Flavobacteriales;f__Flavobacteriaceae;g__Lutaonella-Winogradskyella	0.000121475	0.000155088	0	0
k__Bacteria;p__Bacteroidetes;c__Flavobacteriia;o__Flavobacteriales;f__Flavobacteriaceae;g__Lutibacter	0.000286279	0.000238596	0	0
k__Bacteria;p__Bacteroidetes;c__Flavobacteriia;o__Flavobacteriales;f__Flavobacteriaceae;g__Lutibacter-Tenacibaculum	0	0.000128641	0	0
k__Bacteria;p__Bacteroidetes;c__Flavobacteriia;o__Flavobacteriales;f__Flavobacteriaceae;g__Mariniflexile	0.000247909	0	0	0
k__Bacteria;p__Bacteroidetes;c__Flavobacteriia;o__Flavobacteriales;f__Flavobacteriaceae;g__Muricauda	0.000666139	0.000869876	0	0
k__Bacteria;p__Bacteroidetes;c__Flavobacteriia;o__Flavobacteriales;f__Flavobacteriaceae;g__Olleya	0.003059784	0.0034357	5.67E-05	0
k__Bacteria;p__Bacteroidetes;c__Flavobacteriia;o__Flavobacteriales;f__Flavobacteriaceae;g__Polaribacter	0.000972809	0.001892292	0.000878137	0
k__Bacteria;p__Bacteroidetes;c__Flavobacteriia;o__Flavobacteriales;f__Flavobacteriaceae;g__Polaribacter-Tenacibaculum	0.000347072	0	0	0
k__Bacteria;p__Bacteroidetes;c__Flavobacteriia;o__Flavobacteriales;f__Flavobacteriaceae;g__Pseudofulvibacter	0.006444233	0.005968268	0	0
k__Bacteria;p__Bacteroidetes;c__Flavobacteriia;o__Flavobacteriales;f__Flavobacteriaceae;g__Sediminicola	0.000833779	0.001096886	0	0.000129316
k__Bacteria;p__Bacteroidetes;c__Flavobacteriia;o__Flavobacteriales;f__Flavobacteriaceae;g__Tenacibaculum	0.01349904	0.014126207	0.000141635	0
k__Bacteria;p__Candidatus Tectomicrobia;c__;o__;f__;g__Candidatus Entotheonella;s__Uncultured Candidatus Entotheonella sp.	0.000192342	0.000200003	0	0

k__Bacteria;p__Chlamydiae;c__Chlamydiae;o__Chlamydiales;f__Chlamydiales Incertae Sedis;g__Criblamydia	0	0.000114581	0	0
k__Bacteria;p__Chlamydiae;c__Chlamydiia;o__Chlamydiales;f__Parachlamydiaceae;g__Parachlamydia	0	0.000517264	0	0
k__Bacteria;p__Chlamydiae;c__Chlamydiia;o__Chlamydiales;f__Rhabdochlamydiaceae;g__Candidatus Rhabdochlamydia	0	8.40331E-05	0	0
k__Bacteria;p__Chlamydiae;c__Chlamydiia;o__Chlamydiales;f__Waddliaceae;g__Waddlia	0	0.000517286	0	0
k__Bacteria;p__Cyanobacteria;c__Cyanobacteria;o__SubsectionI;f__FamilyI;g__Prochlorococcus-Synechococcus	0.001452746	0	0	0
k__Bacteria;p__Cyanobacteria;c__Synechococcophycideae;o__Synechococcales;f__Synechococcaceae;g__Prochlorococcus	0	0	0.000963118	0
k__Bacteria;p__Cyanobacteria;c__Synechococcophycideae;o__Synechococcales;f__Synechococcaceae;g__Synechococcus	0.089283318	0.07174683	0.018098125	0.086195526
k__Bacteria;p__Firmicutes;c__Bacilli;o__Lactobacillales;f__Aerococcaceae;g__Aerococcus	0	0	0	0.000129316
k__Bacteria;p__Firmicutes;c__Bacilli;o__Turicibacterales;f__Turicibacteraceae;g__Turicibacter	0	0	0	0.000193974
k__Bacteria;p__Firmicutes;c__Clostridia;o__Clostridiales;f__[Acidaminobacteraceae];g__Fusibacter	0	0	0	0.000129316
k__Bacteria;p__Firmicutes;c__Clostridia;o__Clostridiales;f__[Tissierellaceae];g__Anaerococcus	0	0	0	0.000129316
k__Bacteria;p__Firmicutes;c__Clostridia;o__Clostridiales;f__Clostridiaceae;g__Alkaliphilus	0	0	0	0.000258632
k__Bacteria;p__Firmicutes;c__Clostridia;o__Clostridiales;f__Clostridiaceae;g__Clostridium	0	0	0.000198289	0
k__Bacteria;p__Firmicutes;c__Clostridia;o__Clostridiales;f__Ruminococcaceae;g__Oscillospira	0	0	0	0.000193974
k__Bacteria;p__Fusobacteria;c__Fusobacteriia;o__Fusobacteriales;f__Fusobacteriaceae;g__Propionigenium	0	0	5.67E-05	0
k__Bacteria;p__Planctomycetes;c__Planctomycetacia;o__Planctomycetales;f__Planctomycetaceae;g__Rubripirellula	0.000265263	0	0	0
k__Bacteria;p__Planctomycetes;c__Planctomycetia;o__Planctomycetales;f__Planctomycetaceae;g__Planctomyces	0	0	0.003223387	0.001810423
k__Bacteria;p__Proteobacteria;c__Alphaproteobacteria;o__Kiloniellales;f__Kiloniellaceae;g__Thalassospira	0	0	0.00028327	0

k__Bacteria;p__Proteobacteria;c__Alphaproteobacteria;o__Rhizobiales;f__Beijerinckiaceae;g__Chelatococcus	0	0	0.001104753	0
k__Bacteria;p__Proteobacteria;c__Alphaproteobacteria;o__Rhizobiales;f__Brucellaceae;g__Ochrobactrum	0	0	5.67E-05	0
k__Bacteria;p__Proteobacteria;c__Alphaproteobacteria;o__Rhizobiales;f__Cohaesibacteraceae;g__Cohaesibacter	0	0	0.000311597	0
k__Bacteria;p__Proteobacteria;c__Alphaproteobacteria;o__Rhizobiales;f__Hyphomicrobiaceae;g__Devosia	0	0	0.000339924	0.000387948
k__Bacteria;p__Proteobacteria;c__Alphaproteobacteria;o__Rhizobiales;f__Hyphomicrobiaceae;g__Hyphomicrobium	0.000480943	0.000555214	5.67E-05	0
k__Bacteria;p__Proteobacteria;c__Alphaproteobacteria;o__Rhizobiales;f__Hyphomicrobiaceae;g__Rhodoplanes	0	0	0.000113308	0.000258632
k__Bacteria;p__Proteobacteria;c__Alphaproteobacteria;o__Rhizobiales;f__Methylobacteriaceae;g__Microvirga	0.000111671	0.000192412	0	0
k__Bacteria;p__Proteobacteria;c__Alphaproteobacteria;o__Rhizobiales;f__Methylocystaceae;g__Pleomorphomonas	0	0	5.67E-05	0
k__Bacteria;p__Proteobacteria;c__Alphaproteobacteria;o__Rhizobiales;f__Phyllobacteriaceae;g__Chelativorans	0	0	8.50E-05	0
k__Bacteria;p__Proteobacteria;c__Alphaproteobacteria;o__Rhizobiales;f__Phyllobacteriaceae;g__Hoeflea	0.021850689	0.028663329	0	0
k__Bacteria;p__Proteobacteria;c__Alphaproteobacteria;o__Rhizobiales;f__Phyllobacteriaceae;g__Mesorhizobium	0	0	5.67E-05	0
k__Bacteria;p__Proteobacteria;c__Alphaproteobacteria;o__Rhizobiales;f__Phyllobacteriaceae;g__Nitratireductor	0.000831243	0.001015059	0	0
k__Bacteria;p__Proteobacteria;c__Alphaproteobacteria;o__Rhizobiales;f__Phyllobacteriaceae;g__Phyllobacterium	0	0	0.000538213	0
k__Bacteria;p__Proteobacteria;c__Alphaproteobacteria;o__Rhizobiales;f__Rhizobiaceae;g__Agrobacterium	0	0	0.000736502	0
k__Bacteria;p__Proteobacteria;c__Alphaproteobacteria;o__Rhizobiales;f__Rhizobiaceae;g__Neorhizobium	0.035904445	0.047676717	0	0
k__Bacteria;p__Proteobacteria;c__Alphaproteobacteria;o__Rhizobiales;f__Rhizobiaceae;g__Rhizobium	0	0	0.000368251	0
k__Bacteria;p__Proteobacteria;c__Alphaproteobacteria;o__Rhizobiales;f__Rhodobiaceae;g__Afifella			0	0.000258632
k__Bacteria;p__Proteobacteria;c__Alphaproteobacteria;o__Rhodobacterales;f__Rhodobacteraceae;g__Citreimonas-Phaeobacter	9.17263E-05	0	0	0

k__Bacteria;p__Proteobacteria;c__Alphaproteobacteria;o__Rhodobacterales;f__Rhodobacteraceae;g__Leisingera	0.014471416	0.012327561	0	0
k__Bacteria;p__Proteobacteria;c__Alphaproteobacteria;o__Rhodobacterales;f__Rhodobacteraceae;g__Nautella	0	0	0	0.000193974
k__Bacteria;p__Proteobacteria;c__Alphaproteobacteria;o__Rhodobacterales;f__Rhodobacteraceae;g__Nautella-Ruegeria	0.0002727	0	0	0
k__Bacteria;p__Proteobacteria;c__Alphaproteobacteria;o__Rhodobacterales;f__Rhodobacteraceae;g__Nesiotobacter	0.000303745	0.00025655	0	0
k__Bacteria;p__Proteobacteria;c__Alphaproteobacteria;o__Rhodobacterales;f__Rhodobacteraceae;g__Octadecabacter	0	0	0.001246388	0.000452606
k__Bacteria;p__Proteobacteria;c__Alphaproteobacteria;o__Rhodobacterales;f__Rhodobacteraceae;g__Phaeobacter	0.007739406	0.006468467	0	0
k__Bacteria;p__Proteobacteria;c__Alphaproteobacteria;o__Rhodobacterales;f__Rhodobacteraceae;g__Planktotalea	0.000617293	0	0	0
k__Bacteria;p__Proteobacteria;c__Alphaproteobacteria;o__Rhodobacterales;f__Rhodobacteraceae;g__Ponticoccus	0.000282616	0	0	0
k__Bacteria;p__Proteobacteria;c__Alphaproteobacteria;o__Rhodobacterales;f__Rhodobacteraceae;g__Primorskyibacter	0.000473485	0.000396143	0	0
k__Bacteria;p__Proteobacteria;c__Alphaproteobacteria;o__Rhodobacterales;f__Rhodobacteraceae;g__Pseudodonghicola	0.000366905	0	0	0
k__Bacteria;p__Proteobacteria;c__Alphaproteobacteria;o__Rhodobacterales;f__Rhodobacteraceae;g__Pseudoroseovarius	0.000875118	0.001088716	0	0
k__Bacteria;p__Proteobacteria;c__Alphaproteobacteria;o__Rhodobacterales;f__Rhodobacteraceae;g__Pseudovibrio	0.000425086	0.000633026	0	0
k__Bacteria;p__Proteobacteria;c__Alphaproteobacteria;o__Rhodobacterales;f__Rhodobacteraceae;g__Rhodobaca	0	0	2.83E-05	0
k__Bacteria;p__Proteobacteria;c__Alphaproteobacteria;o__Rhodobacterales;f__Rhodobacteraceae;g__Rhodobacter	0	0	0.000453232	0
k__Bacteria;p__Proteobacteria;c__Alphaproteobacteria;o__Rhodobacterales;f__Rhodobacteraceae;g__Rhodovulum	0	0	0.000141635	0.000129316
k__Bacteria;p__Proteobacteria;c__Alphaproteobacteria;o__Rhodobacterales;f__Rhodobacteraceae;g__Roseobacter	0	0	0	0.000129316
k__Bacteria;p__Proteobacteria;c__Alphaproteobacteria;o__Rhodobacterales;f__Rhodobacteraceae;g__Ruegeria	0.000918604	0.000520645	0.00028327	0.005819216
k__Bacteria;p__Proteobacteria;c__Alphaproteobacteria;o__Rhodobacterales;f__Rhodobacteraceae;g__Sulfitobacter	7.83957E-05	0	0	0

k__Bacteria;p__Proteobacteria;c__Alphaproteobacteria;o__Rhodobacterales;f__Rhodobacteraceae;g__Thioclava	0.000260304	0.000403228	0	0
k__Bacteria;p__Proteobacteria;c__Alphaproteobacteria;o__Rhodospirillales;f__Rhodospirillaceae;g__Inquilinus	0	0	0	0.000258632
k__Bacteria;p__Proteobacteria;c__Alphaproteobacteria;o__Rhodospirillales;f__Rhodospirillaceae;g__Magnetospirillum	0	0	2.83E-05	0
k__Bacteria;p__Proteobacteria;c__Alphaproteobacteria;o__Rickettsiales;f__Anaplasmataceae;g__Candidatus Neoehrlichia	0	0	8.50E-05	0
k__Bacteria;p__Proteobacteria;c__Alphaproteobacteria;o__SAR11 clade;f__Surface 1;g__Pelagibacter	0.000187607	0.000169775	0	0
k__Bacteria;p__Proteobacteria;c__Alphaproteobacteria;o__Sphingomonadales;f__Sphingomonadaceae;g__Sphingomonas	0	0	0	0.000258632
k__Bacteria;p__Proteobacteria;c__Betaproteobacteria;o__Methylophilales;f__Methylophilaceae;g__Methylotenera	0	0	5.67E-05	0
k__Bacteria;p__Proteobacteria;c__Deltaproteobacteria;o__Bdellovibrionales;f__Bacteriovoracaceae;g__Bacteriovorax	0	0	0	0.000133883
k__Bacteria;p__Proteobacteria;c__Deltaproteobacteria;o__Bdellovibrionales;f__Bdellovibrionaceae;g__Bdellovibrio	0	0	0.000341815	5.36E-05
k__Bacteria;p__Proteobacteria;c__Deltaproteobacteria;o__Desulfobacterales;f__Nitrospinaceae;g__Nitrospina	0	0	7.60E-05	0.001044289
k__Bacteria;p__Proteobacteria;c__Deltaproteobacteria;o__Desulfovibrionales;f__Desulfomicrobiaceae;g__Desulfomicrobium	0	0	0	5.36E-05
k__Bacteria;p__Proteobacteria;c__Deltaproteobacteria;o__Desulfovibrionales;f__Desulfovibrionaceae;g__Desulfovibrio	0	0	0	5.36E-05
k__Bacteria;p__Proteobacteria;c__Deltaproteobacteria;o__Myxococcales;f__Myxococcaceae;g__Anaeromyxobacter	0	0	0	5.36E-05
k__Bacteria;p__Proteobacteria;c__Epsilonproteobacteria;o__Campylobacterales;f__Campylobacteraceae;g__Arcobacter	0.093841455	0.111250132	0	0
k__Bacteria;p__Proteobacteria;c__Gammaproteobacteria;o__[Marinicellales];f__[Marinicellaceae];g__Marinicella	0.000620086	0.000471153	0	0
k__Bacteria;p__Proteobacteria;c__Gammaproteobacteria;o__Alteromonadales;f__Alteromonadaceae;g__Agarivorans	0	0	7.60E-05	0
k__Bacteria;p__Proteobacteria;c__Gammaproteobacteria;o__Alteromonadales;f__Alteromonadaceae;g__HB2-32-21	0	0	0.000227877	0.000455203
k__Bacteria;p__Proteobacteria;c__Gammaproteobacteria;o__Alteromonadales;f__Alteromonadaceae;g__Marinobacter	0	0	7.60E-05	0

k__Bacteria;p__Proteobacteria;c__Gammaproteobacteria;o__Alteromonadales;f__Alteromonadaceae;g__Microbulbifer	0	0	2.83E-05	0.00064658
k__Bacteria;p__Proteobacteria;c__Gammaproteobacteria;o__Alteromonadales;f__Alteromonadaceae;g__Spongiibacter	0.002915055	0.003415456	8.50E-05	0
k__Bacteria;p__Proteobacteria;c__Gammaproteobacteria;o__Alteromonadales;f__Colwelliaceae;g__Colwellia	0.001060046	0.001106698	0	0
k__Bacteria;p__Proteobacteria;c__Gammaproteobacteria;o__Alteromonadales;f__Colwelliaceae;g__Thalassomonas	0.000228076	0	0	0
k__Bacteria;p__Proteobacteria;c__Gammaproteobacteria;o__Alteromonadales;f__Colwelliaceae;g__Thalassotalea	0.000228076	0	0	0
k__Bacteria;p__Proteobacteria;c__Gammaproteobacteria;o__Alteromonadales;f__HTCC2188;g__HTCC	0	0	0.000764829	0
k__Bacteria;p__Proteobacteria;c__Gammaproteobacteria;o__Alteromonadales;f__Pseudoalteromonadaceae;g__Pseudoalteromonas	0.001250422	0.000978513	0	0
k__Bacteria;p__Proteobacteria;c__Gammaproteobacteria;o__Alteromonadales;f__Shewanellaceae;g__Psychrobium	0.002386986	0.002402172	0	0
k__Bacteria;p__Proteobacteria;c__Gammaproteobacteria;o__Alteromonadales;f__Shewanellaceae;g__Shewanella	0.002418835	0.002512535	0	0.000969869
k__Bacteria;p__Proteobacteria;c__Gammaproteobacteria;o__Cellvibrionales;f__Cellvibrionaceae;g__Marinimicrobium	0.001625929	0.001693982	0	0
k__Bacteria;p__Proteobacteria;c__Gammaproteobacteria;o__Cellvibrionales;f__Porticoccaceae;g__Porticoccus	0	0.000286732	0	0
k__Bacteria;p__Proteobacteria;c__Gammaproteobacteria;o__Legionellales;f__Coxiellaceae;g__Aquicella	0	0	5.67E-05	0.000711238
k__Bacteria;p__Proteobacteria;c__Gammaproteobacteria;o__Legionellales;f__Coxiellaceae;g__Coxiella	0.003659183	0.003653475	0	0
k__Bacteria;p__Proteobacteria;c__Gammaproteobacteria;o__Legionellales;f__Coxiellaceae;g__Rickettsiella	0	0	0	0.000581922
k__Bacteria;p__Proteobacteria;c__Gammaproteobacteria;o__Legionellales;f__Legionellaceae;g__Legionella	0.003930611	0.003685356	0	0.000517264
k__Bacteria;p__Proteobacteria;c__Gammaproteobacteria;o__Legionellales;f__Legionellaceae;g__Tatlockia	0	0	0	0.00032329
k__Bacteria;p__Proteobacteria;c__Gammaproteobacteria;o__Methylococcales;f__Crenotrichaceae;g__Crenothrix	0	0	0	0.00032329
k__Bacteria;p__Proteobacteria;c__Gammaproteobacteria;o__Methylococcales;f__Methylococcaceae;g__Methylocaldum	0	0	0.000169962	0

k__Bacteria;p__Proteobacteria;c__Gammaproteobacteria;o__Oceanospirillales;f__Alcanivoracaceae;g__Alcanivorax	0.000920927	0.001351258	8.50E-05	0
k__Bacteria;p__Proteobacteria;c__Gammaproteobacteria;o__Oceanospirillales;f__Alcanivoracaceae;g__Kangiella	0.001280283	0.001411698	0	0
k__Bacteria;p__Proteobacteria;c__Gammaproteobacteria;o__Oceanospirillales;f__Hahellaceae;g__Endozoicomonas	0.084329039	0.061244298	0	0
k__Bacteria;p__Proteobacteria;c__Gammaproteobacteria;o__Oceanospirillales;f__Hahellaceae;g__Kistimonas	0.002474628	0.00202215	0	0
k__Bacteria;p__Proteobacteria;c__Gammaproteobacteria;o__Oceanospirillales;f__Halomonadaceae;g__Candidatus Portiera	0	0	0.003795819	0
k__Bacteria;p__Proteobacteria;c__Gammaproteobacteria;o__Oceanospirillales;f__Oceanospirillaceae;g__Amphritea	0.009112877	0.011582935	0	0
k__Bacteria;p__Proteobacteria;c__Gammaproteobacteria;o__Oceanospirillales;f__Oceanospirillaceae;g__Marinobacterium	0.002023989	0.002239745	0	0
k__Bacteria;p__Proteobacteria;c__Gammaproteobacteria;o__Oceanospirillales;f__Oceanospirillaceae;g__Marinomonas	0.00160529	0.001963107	0	0
k__Bacteria;p__Proteobacteria;c__Gammaproteobacteria;o__Oceanospirillales;f__Oceanospirillaceae;g__Motiliproteus	0.000294699	0.000277577	0	0
k__Bacteria;p__Proteobacteria;c__Gammaproteobacteria;o__Oceanospirillales;f__Oceanospirillaceae;g__Neptuniibacter	0.000290053	0.000421839	0	0
k__Bacteria;p__Proteobacteria;c__Gammaproteobacteria;o__Oceanospirillales;f__Oceanospirillaceae;g__Oceanospirillum	0.001289126	0.001721475	0	0
k__Bacteria;p__Proteobacteria;c__Gammaproteobacteria;o__Order Incertae Sedis;f__Family Incertae Sedis;g__Marinicella	0.000620086	0.000471153	0	0
k__Bacteria;p__Proteobacteria;c__Gammaproteobacteria;o__Pseudomonadales;f__Pseudomonadaceae;g__Pseudomonas	0	0	5.67E-05	0
k__Bacteria;p__Proteobacteria;c__Gammaproteobacteria;o__Vibrionales;f__Pseudoalteromonadaceae;g__Pseudoalteromonas	0.000785871	0.000638962	0	0
k__Bacteria;p__Proteobacteria;c__Gammaproteobacteria;o__Vibrionales;f__Vibrionaceae;g__Photobacterium	0.000580107	0.000626555	0	0
k__Bacteria;p__Proteobacteria;c__Gammaproteobacteria;o__Vibrionales;f__Vibrionaceae;g__Vibrio	0.008422347	0.01557361	0	0.000129316
k__Bacteria;p__Proteobacteria;c__Gammaproteobacteria;o__Xanthomonadales;f__Xanthomonadaceae;g__Thermomonas	0	0	0	0.000129316
k__Bacteria;p__Thermi;c__Deinococci;o__Deinococcales;f__Trueperaceae;g__B-42	0	0	2.83E-05	0

k__Bacteria;p__Verrucomicrobia;c__[Spartobacteria];o__[Chthoniobacterales]; f__[Chthoniobacteraceae];g__Candidatus Xiphinematobacter	0	0	0	5.39739E-05
k__Bacteria;p__Verrucomicrobia;c__Verrucomicrobiae;o__Verrucomicrobiales; f__Verrucomicrobiaceae;g__Rubritalea	0	0	2.83E-05	5.78171E-05
k__Bacteria;p__Verrucomicrobia;c__Verrucomicrobiae;o__Verrucomicrobiales; f__Verrucomicrobiaceae;g__Verrucomicrobium	0	0	5.67E-05	2.00159E-05
Unassigned;Other;Other;Other;Other;Other	0.559994839	0.558000245	0.962007144	0.895032089
